# Scalable hydrogen-bonded organic framework-based membranes for efficient ultra-high PFOA adsorption

**DOI:** 10.1039/d6sc03163c

**Published:** 2026-08-11

**Authors:** Zhangjie Gu, Yi Li, Hongbing Li, Yanlei He, Wenjie Wang, Yonggang Yao, Richard J. Hooley, Jonathan L. Sessler, Xiaodong Chi

**Affiliations:** a State Key Laboratory of New Textile Materials and Advanced Processing, School of Materials Science and Engineering, Huazhong University of Science and Technology Wuhan 430074 China xchi@hust.edu.cn; b Department of Chemistry, The University of Texas at Austin Austin Texas 78712-1224 USA sessler@cm.utexas.edu; c Department of Chemistry, University of California–Riverside Riverside CA 92521 USA richard.hooley@ucr.edu

## Abstract

Per- and polyfluoroalkyl substances (PFAS), such as perfluorooctanoic acid (PFOA), pose severe environmental and health risks owing to their persistence and widespread contamination of water resources. Their effective removal from water, however, remains challenging due to the difficulty of simultaneously achieving high removal efficiency, high adsorption capacity, and scalable processability. Here, we address this challenge by integrating high-capacity molecular adsorption directly into a scalable membrane architecture. We report a composite membrane formed by the *in situ* growth of a hydrogen-bonded organic framework (HOF-21m) with exceptional PFAS affinity on a cellulose substrate. The resulting membrane simultaneously delivers 99.9% PFOA rejection and high water permeance (86.3 L m^−2^ h^−1^ bar^−1^), while exhibiting an outstanding PFOA adsorption capacity of (6.57 ± 0.38) × 10^3^ mg g^−1^, enabled by synergistic multi-site interactions within the HOF framework. Importantly, the use of inexpensive ligands and mild synthesis conditions allows roll-to-roll fabrication of large-area continuous membranes (0.6 m × 10 m), demonstrating clear scalability. These findings establish HOF-based membranes as a practical platform for high-performance PFAS removal.

## Abstract 2

Integrating molecular adsorption with membrane separation, a HOF-cellulose composite membrane delivers ultrahigh PFOA uptake, 99.9% rejection, and scalable roll-to-roll manufacturing for practical PFAS remediation.

## Graphical abstract

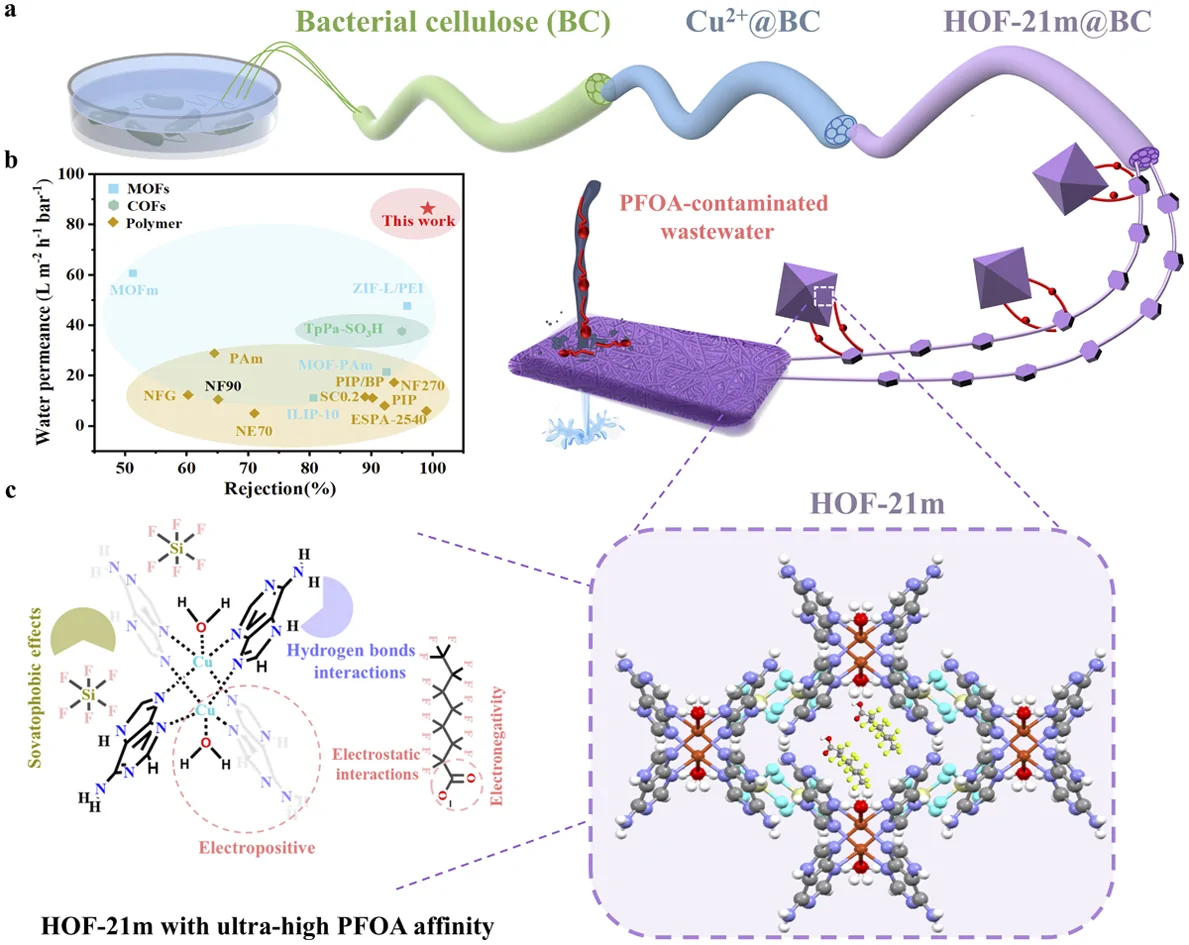

## Introduction

Per- and polyfluoroalkyl substances (PFAS) are a class of highly persistent synthetic compounds that until recently saw extensive use across a variety of industries due to their exceptional thermal and chemical stability.^1^ Unfortunately, their persistence in the environment, bioaccumulation potential, and widespread contamination of water, food, and ecosystems continue to pose significant environmental and health concerns.^2^ Chronic exposure to PFAS has been linked to various health risks, including cancer, thyroid disorders, and metabolic diseases.^3^ Among the PFAS, perfluorooctanoic acid (PFOA) stands out due to its ability to bind to proteins and bioaccumulate in the human body, where it is associated with a wide range of adverse health effects, including kidney and liver damage, immunotoxicity, reproductive toxicity, and cancer.^4^ In recognition of these risks, the U.S. Environmental Protection Agency (US EPA) introduced a recommended limit of 4 ng L^−1^ for PFOA concentrations in drinking water in 2023.^5^ Despite this new guidance, PFOA levels in many water sources, especially those near industrial sites, airports, and military installations, continue to exceed the 4 ng L^−1^ threshold, underscoring the urgent need for effective remediation strategies.

Various strategies have been explored to address PFAS contamination, including electrochemical oxidation,^6,7^ photocatalysis,^8–10^ electrothermal mineralization,^11,12^ biological remediation,^2^ and adsorption.^13–17^ Among these approaches, membrane-based separation^18,19^ and adsorption strategies have attracted attention due to their potential simplicity, operational efficiency, and cost-effectiveness. On the other hand, both these approaches face critical challenges. Reverse osmosis (RO) membranes achieve high PFAS rejection but demand high energy due to dense structures and substantial salt rejection. Conventional nanofiltration (NF) membranes typically remove only 70–90% of PFAS, which is often insufficient to meet drinking water standards. In terms of adsorption, the various adsorbers explored to date, including activated carbon, ion exchange resins, metal organic frameworks (MOFs),^8,20,21^ covalent organic frameworks (COFs),^16,22,23^ porous organic polymers (POPs),^24,25^ and molecular cages,^17^ typically fail to meet practical demands due to their limited affinity, relatively low adsorption capacities and the need for frequent sorbent regeneration or replacement.^24,26^ Moreover, most adsorbents are obtained in powder form, which complicates handling, integration into flow systems, and large-scale deployment. Advanced materials such as MOFs, COFs, and POPs offer improved performance, but their high production costs and complex syntheses remain significant barriers to commercialization.

To address these challenges, we developed a hybrid approach that integrates adsorption and membrane separation into a single platform, combining the high selectivity of porous adsorbents with the operational efficiency and scalability of membrane technology. Central to this strategy is the use of hydrogen-bonded organic frameworks (HOFs), a promising class of crystalline porous materials formed through intermolecular hydrogen bonding between organic or metal–organic building blocks.^27,28^ The modular design of HOFs enables precise control over pore size and chemical functionality,^29,30^ while their dynamic hydrogen-bonding networks allow for solution processability and provide the potential for self-assembly.^31^ These features make HOFs particularly attractive for adsorption applications requiring structural tunability and scalability.^32^

To translate the perceived molecular-level advantages of HOFs into a practical membrane-based PFOA removal system, we developed a cellulose-based composite membrane *via* the *in situ* growth of HOF-21m on cellulose (Scheme 1a). HOF-21m, first reported by Chen's group,^33^ is enriched with fluorine atoms and hydrogen-bonding sites, which collectively enhance PFOA adsorption through electrostatic interactions, hydrogen bonding and the promotion of solvatophobic effects. Cellulose, an abundant, renewable, and biodegradable biopolymer, is widely used in membrane fabrication due to its excellent mechanical strength, hydrophilicity, and chemical tunability.^34,35^ Leveraging these properties, our design not only resolves the conventional trade-off between membrane permeability and contaminant rejection but also enables scalable fabrication using a roll-to-roll strategy, resulting in the fabrication of a large-scale, continuous HOF-21m@cellulose composite membrane (0.6 m × 10 m), as shown in Fig. 4b. As detailed below, the as-synthesized HOF-21m@cellulose composite membrane exhibits outstanding performance, combining high PFOA rejection (99.9%) with high water permeance (86.3 L m^−2^ h^−1^ bar^−1^), and achieves a record-breaking PFOA adsorption capacity of (6.57 ± 0.38) × 10^3^ mg g^−1^. In practical terms for an aqueous sample containing 1500 ng L^−1^, a single treatment resulted in lowering the PFOA level to 61 ng L^−1^. To our knowledge, this work represents the first application of HOF-based materials for PFOA removal and sets a new benchmark for PFAS remediation membranes, while offering a promising, scalable solution for treating contaminated water at large scale.

**Scheme 1:**
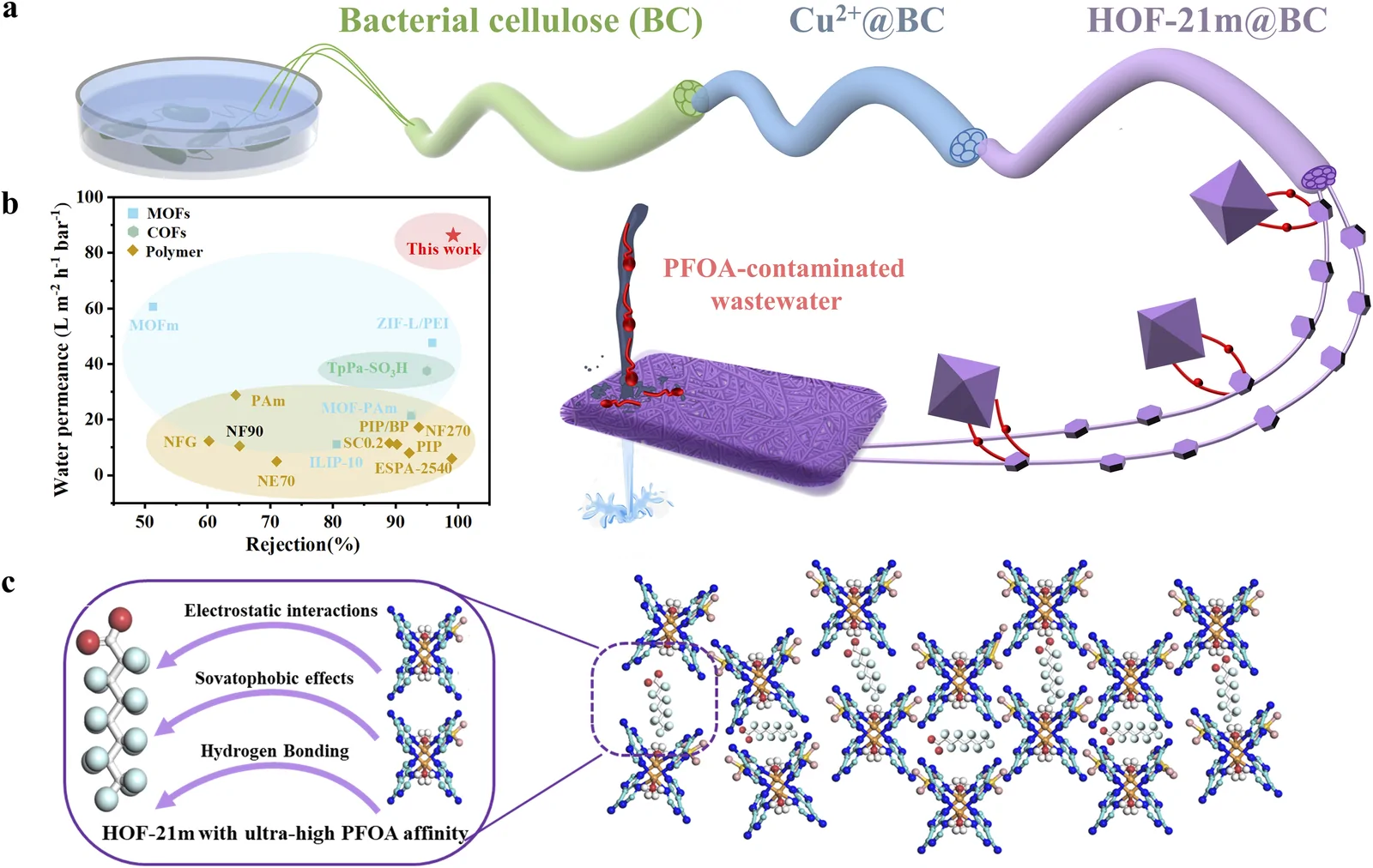
(a) Schematic illustration of HOF-21m@cellulose composite membrane preparation *via in situ* growth. (b) Comparison of PFOA rejection and water permeance achieved using different membranes. For the underlying numerical values and literature references underlying this figure, see Table S3. (c) Cartoon illustration of PFOA capture by HOF-21m.

## Results and discussion

### HOF-21m: preparation, characterization, and PFOA adsorption performance

The HOF-21m@cellulose membrane of this study was expected to combine high water permeance with PFOA rejection. This expectation is predicated on the assumption that HOF-21m would display a high affinity for PFOA. To support this proposition, we first investigated the adsorption behaviour of PFOA. HOF-21m was synthesized according to a previously reported procedure.^33^ Briefly, an acetonitrile–water mixture was layered over a Cu(NO_3_)_2_·3H_2_O/(NH_4_)_2_SiF_6_ solution, followed by dropwise addition of adenine (ade) and, after standing at room temperature for 4 days, yielded blue-black HOF-21 microcrystals (HOF-21m) (Fig. 1a and b). The structure and purity of HOF-21m was confirmed by a variety of characterization techniques, including powder X-ray diffraction (PXRD), Fourier-transform infrared spectroscopy (FT-IR), and scanning electron microscopy (SEM) (Fig. 1c, S2 and S3). Importantly, multiple N–H⋯F hydrogen bonds between [Cu_2_(ade)_4_(H_2_O)_2_]^4+^ and SiF_6_^2−^ confer exceptional water stability on HOF-21m, which is essential for aqueous applications.

**Fig. 1:**
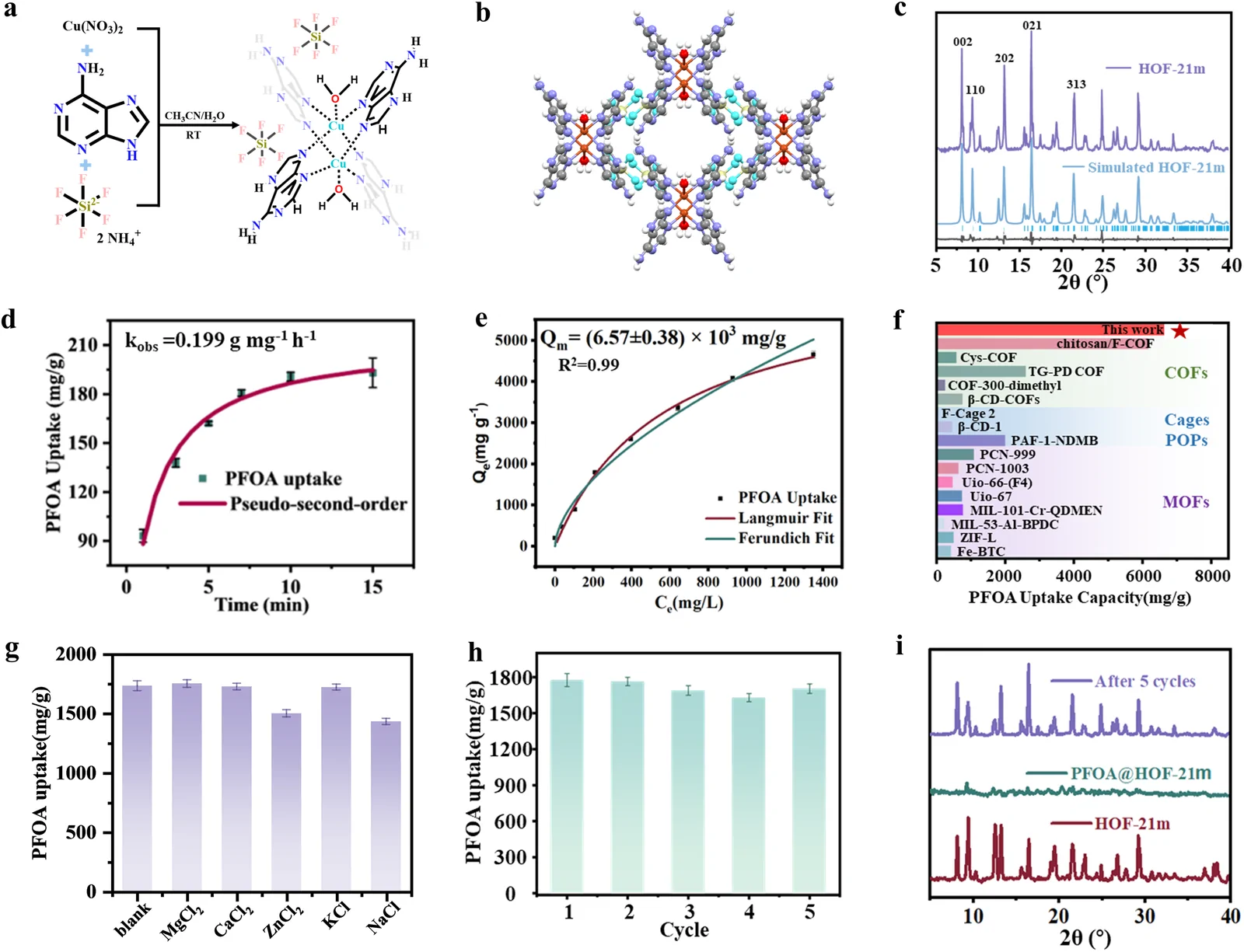
(a) Illustration of the synthetic procedures for HOF-21m. (b) Single-crystal structure of HOF-21m. (c) PXRD pattern confirming the crystallinity of HOF-21m. (d) Adsorption kinetics of HOF-21m for PFOA (200 mg L^−1^), fitted to a pseudo-second-order model. (e) Equilibrium PFOA adsorption isotherms for HOF-21m, fitted to the Langmuir and Freundlich models and derived adsorption capacity (*Q*_m_). (f) Comparison of maximum PFOA *Q*_m_ values for HOF-21m and previously reported adsorbents. (g) PFOA uptake by HOF-21m in the presence of various ionic species. (h) Reusability of HOF-21m for PFOA adsorption over five cycles. (i) PXRD patterns of pristine HOF-21m, PFOA@HOF-21m, and regenerated HOF-21m after five cycles. The error bars represent the standard deviations calculated from measurements of three independent samples in parallel experiments.

To assess its adsorption capacity, activated HOF-21m was immersed in a 2000 mg L^−1^ aqueous PFOA solution. Time-dependent ^19^F NMR measurements (Fig. S4 and S5) show a high PFOA uptake of (1.75 ± 0.32) × 10^3^ mg g^−1^, surpassing most reported PFAS adsorbents, including PCN-999,^20^ TAD-P5 ^25^ and SG-COF-7.^36^ Motivated by this result, we further explored its adsorption kinetics. From an initial 200 mg L^−1^ PFOA solution, HOF-21m was found to remove 93.4% of the PFOA within 7 minutes and 99.2% within 15 minutes (Fig. 1d, S6 and S7). When the adsorption time was extended to 1 h, HOF-21m was able to remove 99.9% of PFOA, giving a final aqueous concentration of 15.9 ng L^−1^ (Fig. S8). The data fits a pseudo-second-order kinetic model with a correlation *R*^2^ = 0.997 and a second-order rate constant *k*_obs_ = 0.199 g mg^−1^ h^−1^. This relatively rapid adsorption is presumably driven by the high porosity of HOF-21m and the multiple binding interactions it provides. Adsorption isotherms were measured with initial PFOA concentrations ranging from 200 to 6000 mg L^−1^ (Fig. S9). The data fitted well to both the Langmuir and Freundlich models (Fig. 1e and Table S1). The maximum capacity predicted by the Langmuir model was (6.57 ± 0.38) × 10^3^ mg g^−1^, setting what to our knowledge is a new record among all known PFOA adsorbents, including MOFs, COFs, POPs, and molecular cages (Fig. 1f and Table S3). This ultrahigh capacity establishes HOF-21m as an attractive material for PFOA remediation.

To evaluate the tolerance of HOF-21m to potential ionic interferants that might be present in typical water samples, HOF-21m was tested in PFOA solutions containing common ions, such as Mg^2+^, Ca^2+^, Zn^2+^, K^+^, Na^+^, and Cl^−^ (Fig. 1g and S10). No significant decrease in PFOA uptake was observed. We thus suggest HOF-21m maintains its adsorption capability in the presence of diverse ionic species and that it could prove amenable to use in complex aqueous environments.

In addition to its high capacity and ion tolerance, HOF-21m also demonstrates favourable cycling stability. Although a substantial reduction in crystallinity is observed after PFOA adsorption, the characteristic PXRD reflections are largely recovered following simple methanol washing (Fig. 1i and S12), indicating reversible structural reorganization of the framework.^37,38^ Consistent with this proposed regeneration behaviour, HOF-21m was found to maintain more than 96% of its initial adsorption capacity after five consecutive adsorption–desorption cycles. SEM imaging further revealed no significant morphological changes after cycling (Fig. S13). Inductively Coupled Plasma Mass Spectrometry (ICP-MS) analysis revealed negligible Cu^2+^ leaching after PFOA adsorption, and the concentration of dissolved Cu^2+^ remained well below the World Health Organization (WHO) guideline value for drinking water (Table S2). Taken in concert, these results highlight the robustness of HOF-21m under conditions that model those relevant to water treatment.

### PFOA adsorption mechanistic study

To elucidate the adsorption mechanism of PFOA by HOF-21m, multiple characterization techniques were employed. FT-IR spectra showed that the adenine N–H bending vibration shifted from 1660 to 1656 cm^−1^, while the SiF_6_^2−^ adsorption band shifted from 1049–933 to 1041–919 cm^−1^ after PFOA adsorption,^39^ indicating strong host–guest interactions (Fig. S12). X-ray photoelectron spectroscopy (XPS) analysis further confirmed successful PFOA uptake as reflected in the increased F 1s signal intensity after exposure to PFOA. High-resolution spectra revealed shifts in the F 1s, N 1s, and Cu 2p signals, reflecting changes in the structural environment and coordination interactions involving Cu sites, respectively (Fig. 2a and S14). CO_2_ adsorption measurements revealed that after PFOA adsorption, the CO_2_ uptake decreased markedly from 35.77 to 9.21 cm^3^ g^−1^ (Fig. 2b). Pore structure analysis indicated a reduction in pore size and a substantial decrease in pore volume from 0.134 to 0.043 cm^3^ g^−1^ (Fig. S15), a result interpreted in terms of substantial occupation of the accessible pores in the network.^20,40^ The ^19^F MAS NMR spectra of PFOA@HOF-21m revealed that the characteristic resonances of PFOA were shifted upon adsorption (*e.g.*, from *δ* = −82.7 and −120.0–−127.2 ppm to −81.3 and −115.7–−126.4 ppm, respectively). In addition, the resonances corresponding to the inner –CF_2_– groups, particularly those adjacent to the carboxylate group, underwent broadening and overlapped. These spectral changes were taken as evidence for the successful incorporation of PFOA into HOF-21m. Furthermore, the spinning sideband intensities arising from ^19^F–^19^F dipolar interactions were markedly reduced after adsorption. This is consistent with restricted molecular mobility and confinement of PFOA within the pores of HOF-21m (Fig. 2c). Moreover, PFOA adsorption promoted the formation of interfacial hydrophobic coatings, which further increased the PFOA uptake (Fig. 2d).^41^

**Fig. 2:**
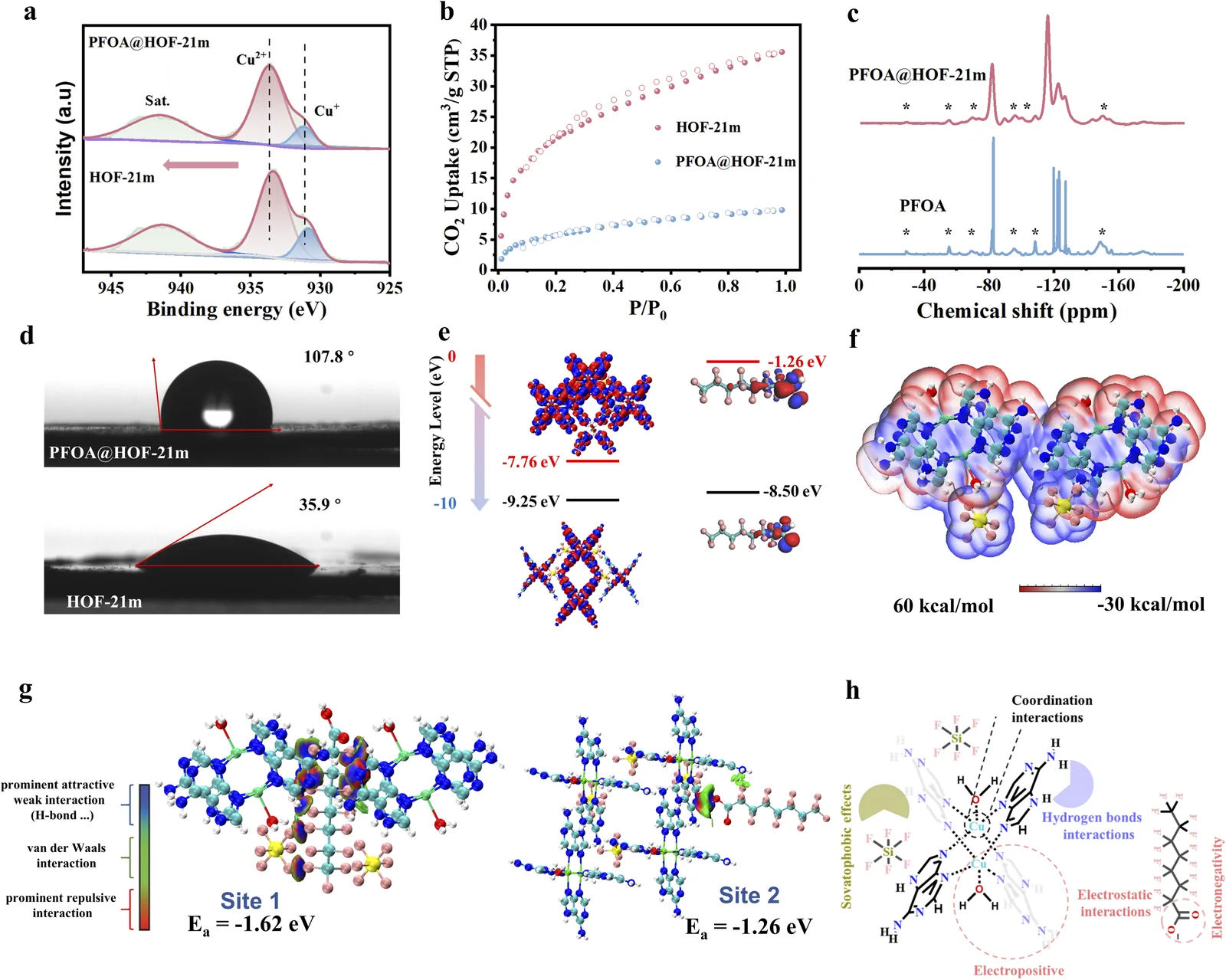
(a) High-resolution Cu 2p of HOF-21m and PFOA@HOF-21m. (b) CO_2_ uptakes of HOF-21m and PFOA@HOF-21m at 298 K. (c) ^19^F MAS NMR spectra of PFOA and PFOA@HOF-21m. Peaks marked by * represent sidebands. (d) Water contact angle of HOF-21m and PFOA@HOF-21m. (e) HOMO and LUMO of a model HOF-21m fragment and PFOA. (f) Molecular electrostatic potential (MESP) distribution of the HOF-21m model compound. (g) Contour plots for an independent gradient model based on Hirshfeld partition (IGMH) isosurfaces illustrating interactions between HOF-21m and PFOA at two different adsorption sites. (h) Illustration of various putative interactions between HOF-21m and PFOA.

To probe the binding environment in more detail, density functional theory (DFT) calculations were carried out using representative molecular fragments of HOF-21m. Frontier molecular orbital analysis (FMO) revealed that the LUMO of HOF-21m are distributed around the Cu-containing and hydrogen-bonding sites (Fig. 2e), leading us to suggest that the Cu-containing and hydrogen-bonding sites act as strong electron acceptors. Molecular electrostatic potential (MESP) maps further revealed that the –NH_2_ and N–H groups of the purine units, together with Cu^2+^-coordinated water molecules (Fig. 2f), are positively polarized and act as polarized hydrogen bond donors. In contrast, the carbonyl oxygen of PFOA functions as a hydrogen-bond acceptor, with its electron density shifting toward these positively polarized sites. Given the abundance of protonation sites (*e.g.*, –NH, –NH_2_) within the HOF-21m framework, protonation readily occurs upon exposure to the protons generated during PFOA dissolution. Consistent with this, zeta potential measurements revealed an approximately sevenfold increase in the positive surface charge density of protonated HOF-21m relative to the non-protonated version (Fig. S16). Consequently, we suggest that electrostatic interactions between HOF-21m and PFOA play a key role in facilitating adsorption.

To identify plausible interaction motifs, two representative host–guest configurations were constructed (Fig. 2g and S18). Site 1 exhibited a lower binding energy and a higher density of active sites compared to Site 2, indicating a more favorable interaction between HOF-21m and PFOA. IGMH analysis revealed multiple non-covalent interactions.^42,43^ Additionally, dipole–dipole interactions between the nitrogen atom in the =N– group and the C–F fluorine atom are thought to enhance PFOA adsorption.^44^ Collectively, these results provide support for the conclusion that the efficient adsorption of PFOA by HOF-21m arises from multiple synergistic interactions, including electrostatic effects, hydrogen bonding interactions, and solvatophobic effects (Fig. 2h).

### PFOA adsorption performance of HOF-based membranes

To develop HOF-21m for practical use, we prepared a composite membrane (HOF-21m@BC) *via in situ* growth on bacterial cellulose (BC) membranes. As shown in Fig. S19, BC membranes were first treated with Cu^2+^ to form Cu^2+^@BC, followed by immersion in a solution of adenine and (NH_4_)_2_SiF_6_ (ACN/H_2_O = 1 : 1), enabling direct crystallization of HOF-21m on the membrane. The resulting membranes were washed and dried for use. Successful integration of HOF-21m into the BC was confirmed by SEM, which showed dense particle coverage on the BC (Fig. 3a and S20). Moreover, EDX mapping revealed a uniform distribution (Fig. S21). PXRD and FT-IR analyses revealed characteristic signals from both HOF-21m and BC (Fig. 3b and c). HOF-21m incorporation slightly increased the membrane thickness and significantly enhanced its mechanical strength (Fig. S22), presumably as the result of hydrogen bonding and network reinforcement (Fig. 3d).^45,46^

**Fig. 3:**
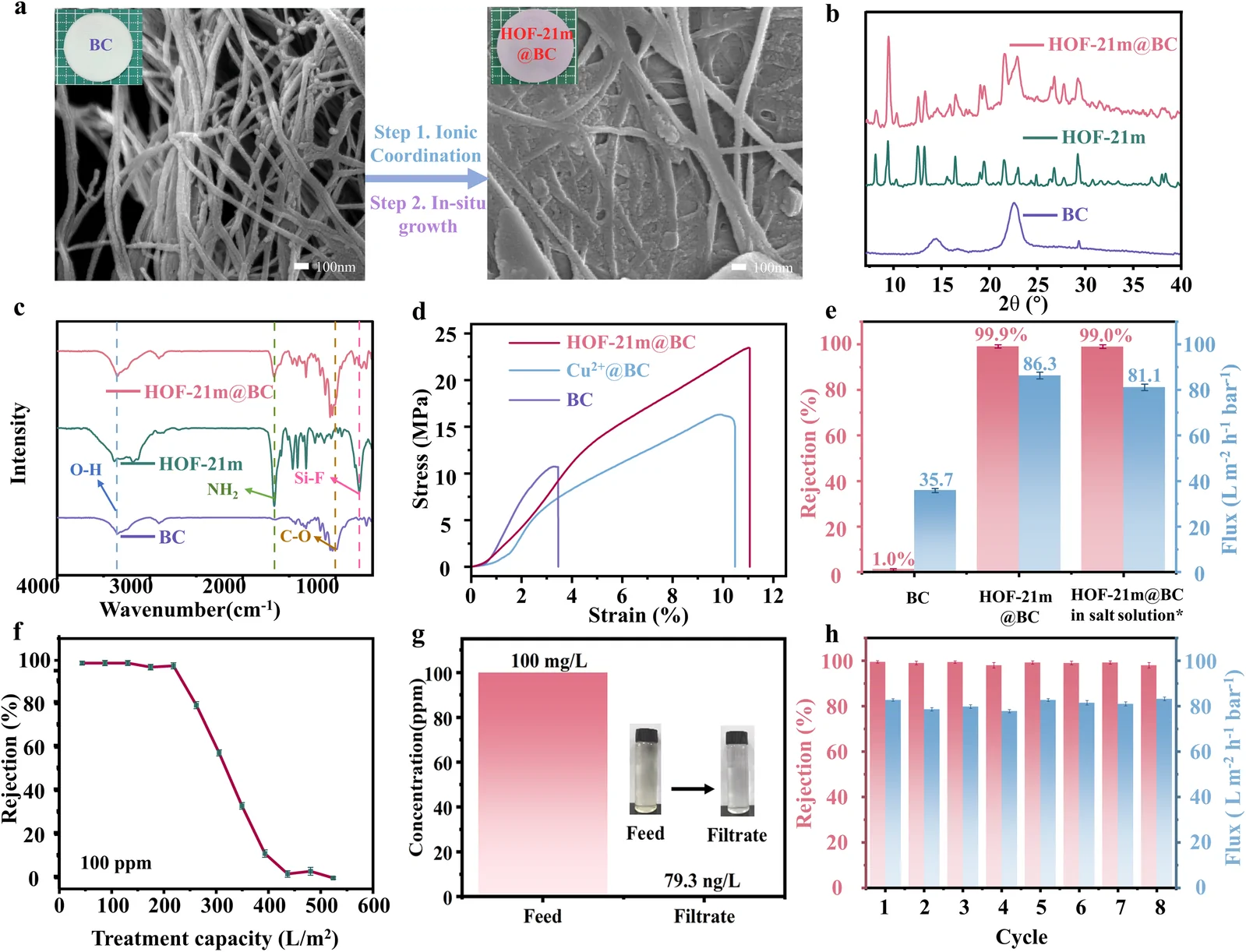
(a) SEM images of pristine bacterial cellulose (BC) and HOF-21m@BC composite membranes. The inset shows photographs of BC, and the HOF-21m@BC membrane of the present study. (b) PXRD spectra of BC, HOF-21m, and HOF-21m@BC. (c) FT-IR spectra of BC, HOF-21m, and HOF-21m@BC. (d) Stress–strain curves for BC, Cu^2+^@BC, and HOF-21m@BC. (e) PFOA rejection and water flux seen for BC, HOF-21m@BC, and HOF-21m@BC in the presence of salt solutions (PFOA concentration of 100 mg L^−1^ and mixed salt solutions of MgCl_2_, CaCl_2_, ZnCl_2_, KCl, and NaCl at concentrations of 0.05 mM, respectively). (f) Breakthrough study of HOF-21m@BC using a 100 mg L^−1^ PFOA solution. (g) PFOA concentrations before and after filtration through HOF-21m@BC. The inset shows photographs of a lake water sample containing PFOA before and after filtration. Note: because the native PFOA concentration in East Lake was relatively low, PFOA was spiked into the lake water to obtain an initial concentration of 100 mg L^−1^ for the adsorption experiments. (h) PFOA rejection and water flux of HOF-21m@BC over multiple filtration–regeneration cycles. The error bars represent the standard deviations calculated from measurements of three independent samples in parallel experiments.

Flow-through experiments using PFOA solutions were then carried out to test the adsorption features of HOF-21m@BC. While a pristine BC membrane exhibits negligible separation capability (1.0% PFOA rejection, 35.7 L m^−2^ h^−1^ bar^−1^ water permeance), the incorporation of HOF-21m transforms its performance. The resulting HOF-21m@BC composite achieves near-complete PFOA rejection (99.9%) and a substantially higher water flux (86.3 L m^−2^ h^−1^ bar^−1^) (Fig. 3e, S23 and S24), outperforming the vast majority of previously reported membrane materials (Scheme 1b and Table S4). This remarkable enhancement stems from the strong adsorption affinity of HOF-21m and the efficient capillary channels and porous framework it introduces.^47^

Subsequently, the effect of HOF-21m loading on the performance of the HOF-21m@BC membrane was investigated. As shown in Fig. S25 and S26, increasing the HOF-21m loading led to a gradual enhancement in PFOA rejection efficiency, followed by a plateau at higher loadings. In contrast, the water flux initially increased and then decreased. This behaviour can be attributed to the increased density of active adsorption sites and the formation of additional capillary transport pathways at moderate loadings, whereas excessive HOF-21m aggregation partially blocks membrane pores and hinders water transport.^48,49^ Importantly, under conditions simulating water matrices with dissolved ions (Mg^2+^, Ca^2+^, Zn^2+^, K^+^, Na^+^, Cl^−^), HOF-21m@BC maintained both high PFOA rejection and effective water flux. Dynamic breakthrough experiments were conducted to evaluate the practical performance of HOF-21m@BC under continuous operation using a 100 mg L^−1^ PFOA solution (Fig. 3f and S27). To normalize membrane area effects, treatment capacity was defined as the treated volume per HOF-21m@BC membrane area. Initially, the membrane achieved 99.2% PFOA rejection; however, performance diminished after 218.3 L m^−2^ with full breakthrough being seen at 438.7 L m^−2^ (Fig. 3f).

To assess the real-world applicability of HOF-21m@BC, the membrane was tested using a PFOA-contaminated lake water sample containing approx. 100 mg L^−1^ PFOA. After filtration, the previously turbid water became visibly clear (Fig. S28). Quantification revealed that over 99.9% of the PFOA was removed to give a final concentration of 79.3 ng L^−1^ (Fig. 3g, S29 and S30). The HOF-21m@BC membrane also showed excellent cycling stability when challenged with lake water over eight adsorption–desorption cycles (Fig. 3h and S31). In these tests, regeneration was carried out *via* simple methanol washing, which was found to preserve both the PFOA rejection capacity and water permeance. Structural integrity was also retained throughout this cycling, as confirmed by consistent PXRD and FT-IR spectral patterns (Fig. S32 and S33) and unchanged SEM images (Fig. S34) reflecting HOF-21m particles firmly retained on the surface. Collectively, these findings lead us to suggest that HOF-21m could have a role to play in removing PFOA from complex aqueous environments.

### Scale-up preparation of membranes *via* a roll-to-roll process

To enable scalable production of HOF-21m@cellulose membranes, we developed a continuous roll-to-roll fabrication process operable under mild conditions (Fig. 4a). Briefly, a cellulose membrane roll was mounted on a rotating drum and passed sequentially through two immersion baths. The first bath contained a 250 mM Cu^2+^ solution and was designed to provide cations that would coordinate to the cellulose hydroxyl groups. The second batch contained adenine and (NH_4_)_2_SiF_6_ (ACN/H_2_O = 1 : 1) and was intended to promote the *in situ* growth of HOF-21m. In accord with these design expectations, after drying, a continuous HOF-21m@cellulose membrane was obtained (Fig. 4b and S35a). SEM imaging confirmed the uniform lamellar deposition of HOF-21m particles along the cellulose fibers (Fig. S35b), while EDX mapping revealed the homogeneous distribution of key constituent elements (Fig. S36). Separately, performance consistency was validated by testing randomly selected samples from the fabricated roll. These various membrane samples exhibited PFOA rejection and structural properties comparable to those made *via* batch synthesis (Fig. 4c and S37). On this basis, we conclude that this roll-to-roll fabrication process allows for the successful integration of HOF-21m into the membrane matrix.

**Fig. 4:**
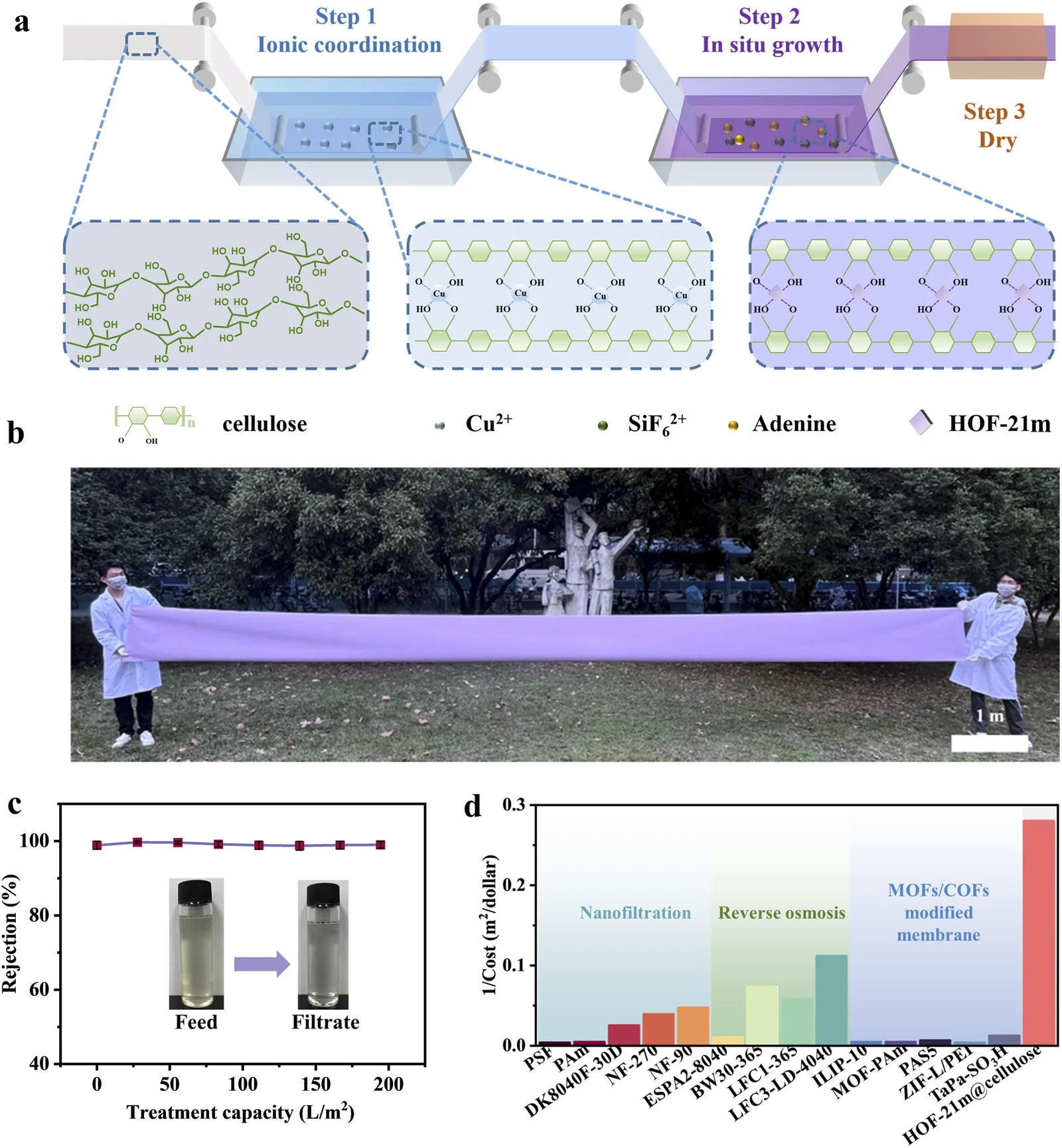
(a) Schematic illustration of a roll-to-roll process that allows for the scalable *in situ* growth of HOF-21m on cellulose membranes. (b) Photograph showing a large-scale HOF-21m@cellulose membrane fabricated by roll-to-roll processing (10 m × 0.6 m). (c) PFOA rejection performance of the HOF-21m@cellulose composite membrane module using a 100 mg L^−1^ PFOA solution prepared with lake water from East Lake, Wuhan. The inset shows pictures of a lake water sample containing PFOA before and after filtration. Note: because the native PFOA concentration in East Lake was relatively low, PFOA was spiked into the lake water to obtain an initial concentration of 100 mg L^−1^ for the adsorption experiments. (d) Comparative material cost analysis of nanofiltration, reverse osmosis, and MOF/COF-modified membranes. The error bars represent the standard deviations calculated from measurements of three independent samples in parallel experiments.

To obtain rough insights into the economics of HOF-21m@cellulose-based PFOA removal, we compared the material and process costs of common alternatives, including nano-filtration, reverse osmosis, and MOF/COF-modified membranes (Fig. 4d and Table S5). While a full comparative cost analysis was limited by the incomplete information in the literature, using estimated pricing for various membrane materials, we conclude that HOF-21m@cellulose represents a relatively low-cost alternative to current approaches. Additionally, it appears to benefit from milder fabrication conditions and higher water permeance than conventional membranes.^50,51^ This is expected to translate into a lower energy demand during both synthesis and operation.

## Conclusions

In conclusion, we developed a high-performance composite membrane by integrating a record-capacity hydrogen-bonded organic framework (HOF-21m) into cellulose. Through the synergy of multi-site molecular interactions and robust membrane architecture, the resulting HOF-21m@BC membrane displayed outstanding PFOA removal, coupled with high water flux and mechanical strength. To enable practical use, a scalable roll-to-roll fabrication strategy was developed that operates under mild conditions. This allowed the continuous production of large-area membranes. The membranes prepared in this way maintain high rejection efficiency, structural integrity, and reusability, even under conditions designed to mimic real-world use. By addressing the limitations of adsorption capacity, stability, and scalability, the HOF-21m@cellulose membranes of this study provide a promising and seemingly cost-effective solution for large-scale PFAS remediation, while highlighting the role porous organic materials are likely to play in environmental remediation applications.

## Author contributions

X. C., R. J. H., and J. L. S. conceived the project, supervised the project, and wrote the manuscript. Y. Y. Y. L. and Z. G. performed the figures visualization. Z. G. and Y. L. performed the experiments and measurements. H. L. performed the PXRD measurements. Y. H. performed the simulation of structure. W. W. performed the SEM measurements. All authors discussed the results and edited the manuscript.

## Conflicts of interest

The authors declare no competing financial interest.

## Supplementary Material

SC-017-D6SC03163C-s001

## Data Availability

The data supporting this article have been included as part of the supplementary information (SI). Supplementary information: experimental methods, synthetic procedures, ^19^F NMR spectra, UPLC-ESI-MS data, FT-IR spectra, SEM images and EDX elemental mapping, and additional characterization data. See DOI: https://doi.org/10.1039/d6sc03163c.
