# Supplementary material for: Scalable hydrogen-bonded organic framework-based membranes for efficient ultra-high PFOA adsorption
Source: Chem Sci. 2026 Aug 11;17(39):18732–40. doi: 10.1039/d6sc03163c (PMC13482906; doi:10.1039/d6sc03163c)
Supplement: Supplementary file 1 [file SC-017-D6SC03163C-s001.pdf]

Supplementary Information (SI) for Chemical Science.
This journal is © The Royal Society of Chemistry 2026

Supporting Information

Scalable Hydrogen-Bonded Organic Framework-based Membranes for Efficient Ultra-High PFOA Adsorption

Zhangjie Gu[1,#], Yi Li[1,#], Hongbing Li[1], Yanlei He[1], Wenjie Wang[1], Yonggang Yao[1], Richard J. Hooley[3,*], Jonathan L. Sessler[2,*] and Xiaodong Chi[1,*]

[1] State Key Laboratory of New Textile Materials and Advanced Processing, School of Materials Science and Engineering, Huazhong University of Science and Technology, Wuhan 430074, China

[2] Department of Chemistry, The University of Texas at Austin, Austin, Texas 78712-1224, United States

[3] Department of Chemistry, University of California–Riverside, Riverside, CA 92521, USA

* Email: richard.hooley@ucr.edu; sessler@cm.utexas.edu; xchi@hust.edu.cn

Experimental Section

1. Materials and methods

Unless otherwise stated, all reagents and solvents were obtained from commercial sources and used without further purification. The bacterial cellulose (BC) membrane used in this study was purchased from the Naimixin Biotechnology Co., Ltd (Shandong). Cellulose membrane was purchased from the Wood Spirit Biotechnology Co., LTD (Tianjin) and used as received. The lake water sample collected from East lake, Wuhan, contained approx. 84.9 ng/L PFOA. Aqueous solutions of perfluorooctanoic acid (PFOA) were prepared using deionized water.

One-dimensional nuclear magnetic resonance (NMR) spectra were recorded on a Bruker AV400 spectrometer. Chemical shifts are quoted in ppm relative to TMS, which was used as an internal reference. The solvent signals were assigned according to He et al.[1] All chemical shifts (δ) are quoted in ppm. Data processing was performed using the MestReNova software. Note: Quantitative ^{19}F NMR analysis is well suited for high-concentration adsorption studies but has limited sensitivity for trace-level PFOA analysis. Therefore, ultra-performance liquid chromatography coupled with electrospray ionization mass spectrometry (UPLC-ESI-MS) is recommended for accurate quantification at environmentally relevant low concentrations due to its significantly higher sensitivity. The ^{19}F MAS NMR experiments were carried out with a Bruker Avance-600 solid-state NMR spectrometer equipped with a standard two-channel 4-mm MAS probe head. Powder X-ray diffraction (PXRD) data were collected on an SmartLab-SE (Rigaku Japan) operating at 40 kV/45 mA using the Cu K_α line (λ = 1.5406 Å). Data were measured over the 5−45° range in 3°/min steps over 14 min. Single crystal X-ray diffraction data were collected on a Rigaku XtaLAB PRO MM007HF X-ray diffractometer with graphite monochromated Cu K_α radiation. CO_2 adsorption measurements were performed at 298 K using ASAP 2020, Micromeritics Instrument Corp, USA. The HOFs were degassed at 25 °C for 12 hours using an automatic volumetric adsorption apparatus. X-ray photoelectron spectroscopy studies were carried out using an AXIS-ULTRA DLD-600W instrument. Scanning electron microscopy (SEM) and energy-dispersive X-ray spectroscopy (EDX) investigations were carried out using a Nova NanoSEM 450 instrument. FTIR spectra were recorded as KBr pellets on a NEXUS 670 FTIR spectrometer, The Zeta potential was measured using a HORIBA SZ-100Z. Quantification of PFOA from the corresponding absorption study data was performed by means of an Ultimate 3000 UPLC-ESI-MS instrument (ThermoFisher, Germany).

Synthesis of HOF-21m

HOF-21 microcrystals (HOF-21m) was prepared using a literature procedure.[2] Briefly, 0.152 mmol (20.5 mg) of adenine dissolved in 3 mL of 1:1 acetonitrile/H_2O at room temperature was layered above 3 mL of an aqueous solution containing 0.076 mmol (17.75 mg) of $Cu(NO_3)_2·3H_2O$ and 0.076 mmol (13.53 mg) of $(NH_4)_2SiF_6$. 1 mL of 1:1 acetonitrile/ H_2O was layered between the top and bottom solutions to slow the rate of reaction. Dark blue, rectangular prismatic crystals formed in 30% yield after 4 days.

Synthesis of HOF-21m@BC

HOF-21m@BC was synthesized in two steps. Firstly, the BC membrane was immersed in 0.25 M $Cu(NO_3)_2·3H_2O$ solution for 1 h, washed with water, and dried at room temperature. This gave a product termed Cu^{2+}@BC. Cu^{2+}@BC was immersed in a mixture of $(NH_4)_2SiF_6$ and adenine

(ACN/H_2O = 1:1) solution for 12 h at room temperature, washed with water, and dried at room temperature to give HOF-21m@BC.

PFOA solution uptake experiments

Kinetic experiments were performed using a stock PFOA solution and HOF-21m at room temperature. The PFOA solution (initial concentration: 2000 ppm) was added to glass vials, and the presumed HOF-21m adsorbent (concentration 1 mg/mL) was added to each vial to form a suspension. Each suspension was continuously stirred until the samples were analyzed at predetermined times (1 min, 3 min, 5 min, 7 min, 10 min, and 15 min). Each aliquot was filtered and a subsample was transferred to an autosampler vial for analysis. The supernatant was analyzed by ^{19}F NMR spectroscopy using trifluoroacetic acid as the internal standard. All batch kinetic experiments were performed in triplicate. The efficiency of PFOA removal (%) by HOF-21m was determined using the following equation:

$$Pollutant\ Removal\ Efficiency = \frac{c_0 - c_t}{c_0} \times 100\%$$

where c_0 (mg/L) and c_t (mg/L) are the concentrations of PFOA before and after adsorption, respectively.

Adsorption kinetic experiments

The amount of adsorbed PFOA was determined using the following equation:

$$q_t = \frac{c_0 - c_t}{c_A}$$

where q_t (mg/g) is the amount of PFOA per g of adsorbent adsorbed at time t (h). c_0 (mg /L) and c_t (mg/L) are the initial and residual concentrations of PFOA in the stock solution and filtrate at time t (h), respectively. c_A (mg/L) is the concentration of the adsorbent.

The adsorption rate of an adsorbent can be described using the pseudo-second-order adsorption the model developed by Ho and McKay,[3] with the linearized form being shown in the following Equation:

$$\frac{t}{q_t} = \frac{t}{q_e} + \frac{1}{k_{obs} q_e^2}$$

where q_e (mg/g) is the amount of PFOA adsorbed at equilibrium and k_{obs} (g mg^{-1} h^{-1}) is an apparent second-order rate constant. The rate constant k_{obs} can be calculated from the intercept and slope of the plot of $\frac{t}{q_t}$ against t.

Isotherm adsorption experiments

Batch isotherm experiments were conducted in 5 mL brown glass vials with magnetic stir bars. The experiments were conducted at 25 °C with magnetic stirring at a stirring rate of 800 revolutions per minute (rpm). PFOA solutions (5 mL; 200, 500, 1000, 2000, 3000, 4000, and 5000 mg/L) were added to brown glass vials before HOF-21m (5 mg) was added to each vial to form a suspension. The suspensions were constantly oscillated for 2 h to allow equilibrium to be reached, before being

filtered through 0.22 μm inorganic membrane filters. The filtrate was analyzed by ^{19}F NMR. All batch experiments were performed in triplicate.

The experimental data was fitted nonlinearly to the Langmuir and Freundlich isotherm models as follows:

$$\frac{1}{q_e} = \frac{1}{Q_m} + \frac{1}{c_e Q_m K_L}$$

where q_e (mg/g) is the amount of pollutant adsorbed at equilibrium, Q_m (mg/g) is the maximum adsorption capacity of the adsorbent at equilibrium, c_e (mg/L) is the residual pollutant concentration at equilibrium, K_L (L/mg) is the equilibrium constant. In the equation below, K_F (mg/g) (L/mg)$^{1/n}$ is the Freundlich constant, and n is an indicator of the intensity of the adsorption.

$$q_e = K_F c_e^{1/n}$$

Membrane separation performance evaluation

The separation performance of all membranes was tested using a dead-end filtration process. Before the test, membranes were pre-pressurized at 3.0 bar for 30 minutes to achieve a steady water flux. Filtration tests were then carried out with feed aqueous solutions containing a specific concentration of PFOA or potentially competing salts at 25 °C and 1.0 bar. Water permeance (P, L $m^{-2} h^{-1}$ per bar) and solute rejection (R, %) were determined using the below equations:

$$P = \frac{V}{A_m \times t \times \Delta P}$$

$$R\ (\%) = \left(1 - \frac{C_p}{C_f}\right) \times 100\%$$

where V is the volume of permeate (l), A_m is the effective membrane area (m^2), t is the filtration time (h), ΔP is the operating pressure (bar), and C_p and C_f are permeate and feed concentrations, respectively. The concentration of PFOA was determined using ^{19}F NMR spectroscopy with trifluoroacetic acid as an internal standard.

DFT calculations

Density functional theory (DFT) calculations were performed with the Gaussian 16 software package. The molecular orbital levels, including the highest occupied molecular orbital (HOMO) and the lowest unoccupied molecular orbital (LUMO), were investigated at the B3LYP-D3/TZVP level of theory. The type of interaction force calculations were carried out using the Multiwfn 3.8 programs.[4]

Membrane Cost Analysis

The price of HOF-21m was \$0.04 per gram, the loading of HOF-21m@cellulose was 100%. Common filter paper could be used as the cellulose membrane, which was priced at \$0.47 per square meter. Thus, the price of HOF-21m@cellulose membrane was calculated \$3.55 per square meter. A full comparative cost analysis was limited by the incomplete information in the literature. However, the material costs of various membranes could be calculated roughly. Subject to this caveat, a cost comparison of various membrane materials was made with the results shown in **Table S4**.

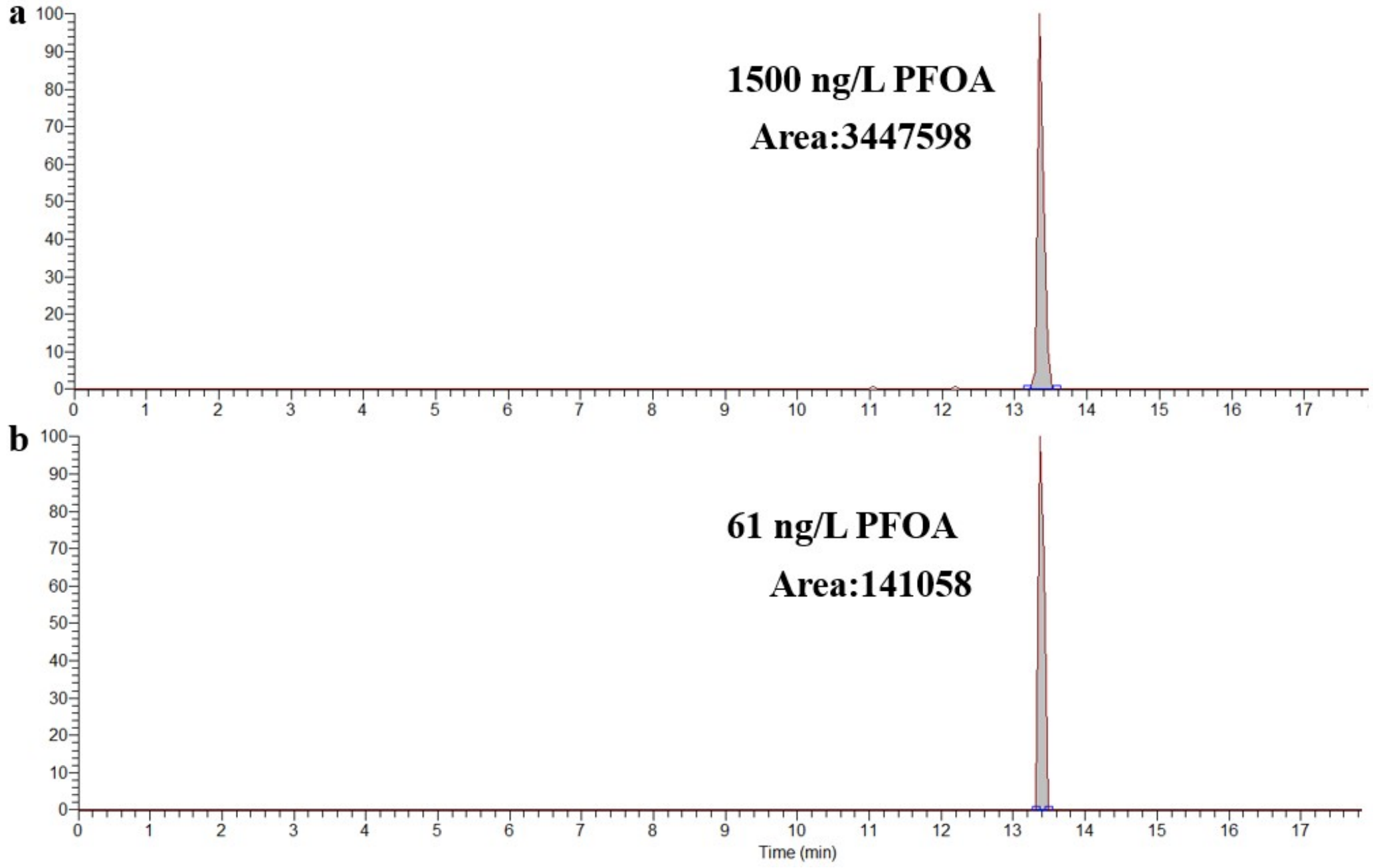

Figure S1. (a) Chromatogram of the initial PFOA solution (1500 ng/L). (b) Chromatogram of the HOF-21m@BC membrane after filtering a 1500 ng/L PFOA solution once. Note: The residual PFOA concentration remains above the current U.S. EPA drinking water guideline level (4 ng/L), indicating that further optimization would be required for applications targeting compliance with such stringent drinking water standards.

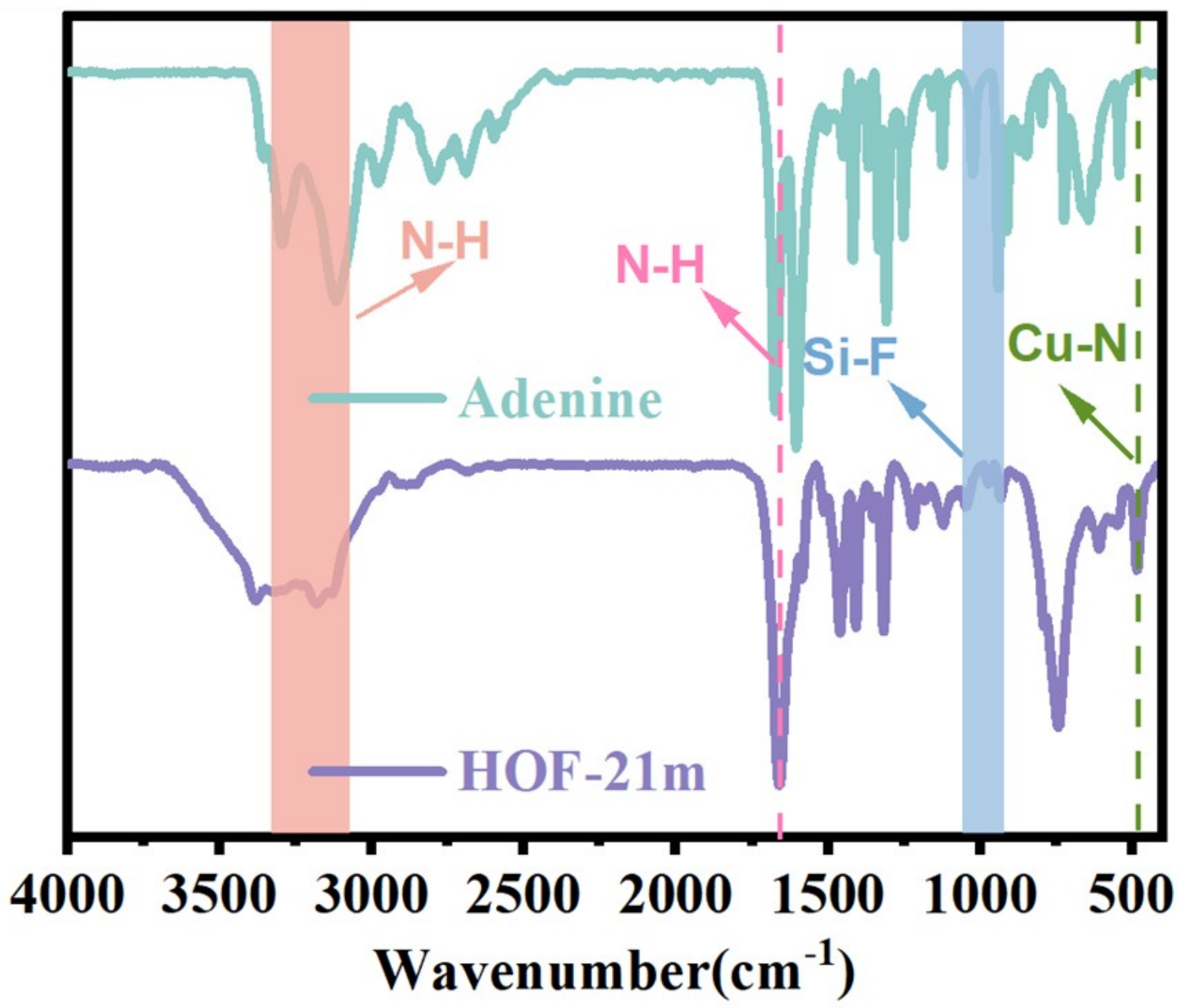

Figure S2. FT-IR spectra of HOF-21m and adenine.

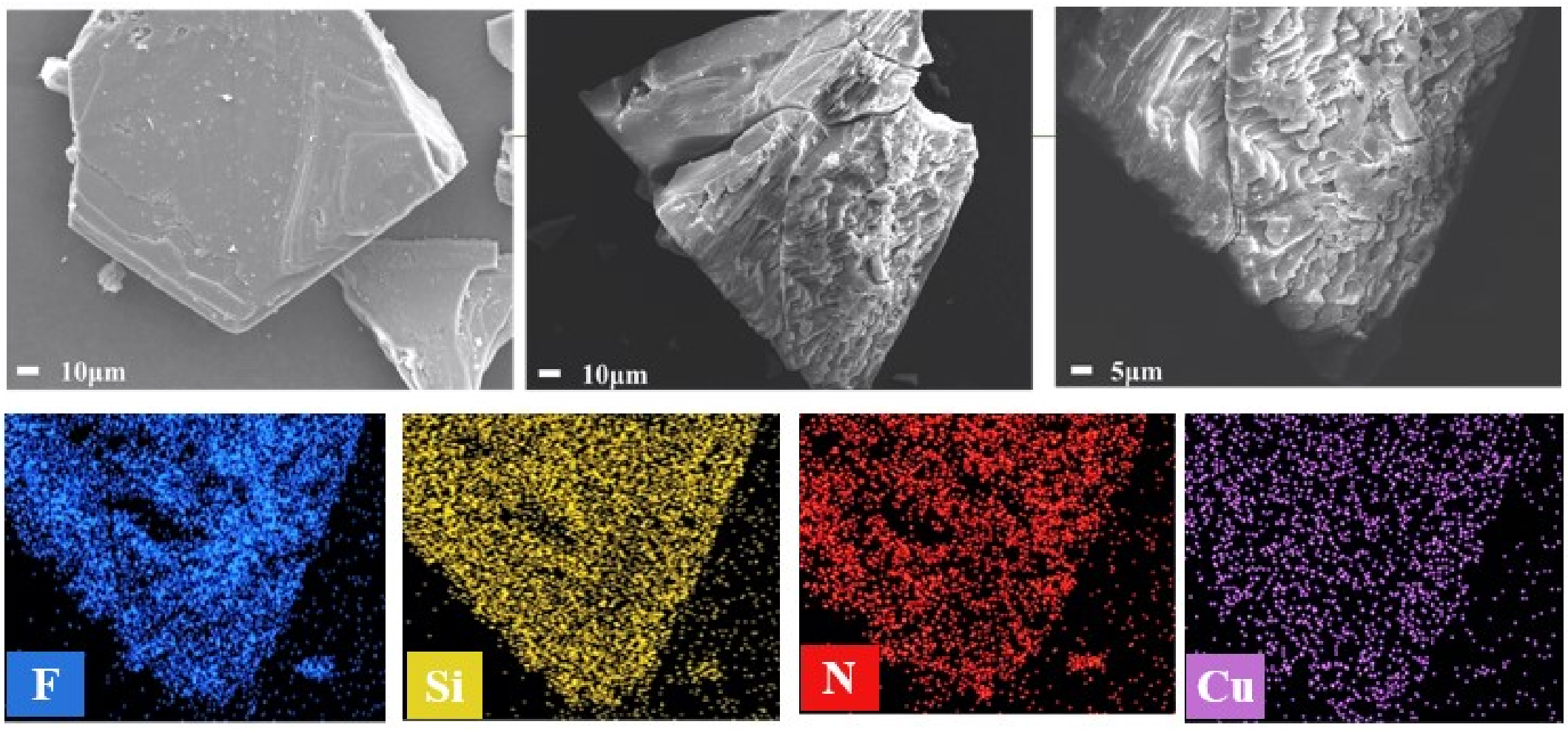

Figure S3. SEM images and EDX mapping of HOF-21m.

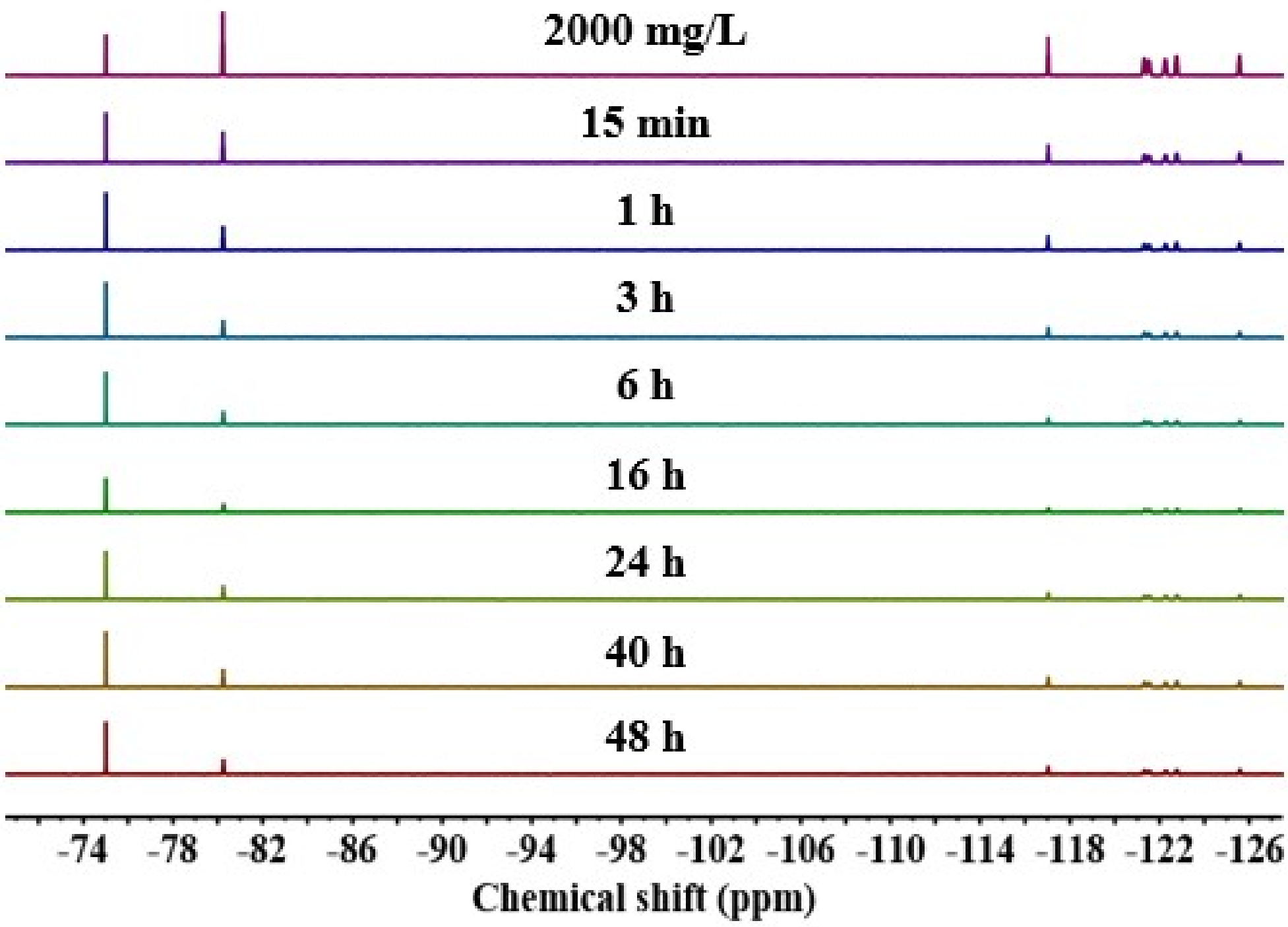

Figure S4. ^{19}F NMR spectra (396 MHz, 298K, H_2O:D_2O = 9:1) of PFOA solution adsorbed by HOF-21m (5 mg) for different times (PFOA solutions 5 mL: 2000 mg/L). Note: Trifluoroacetic acid was used as an internal standard.

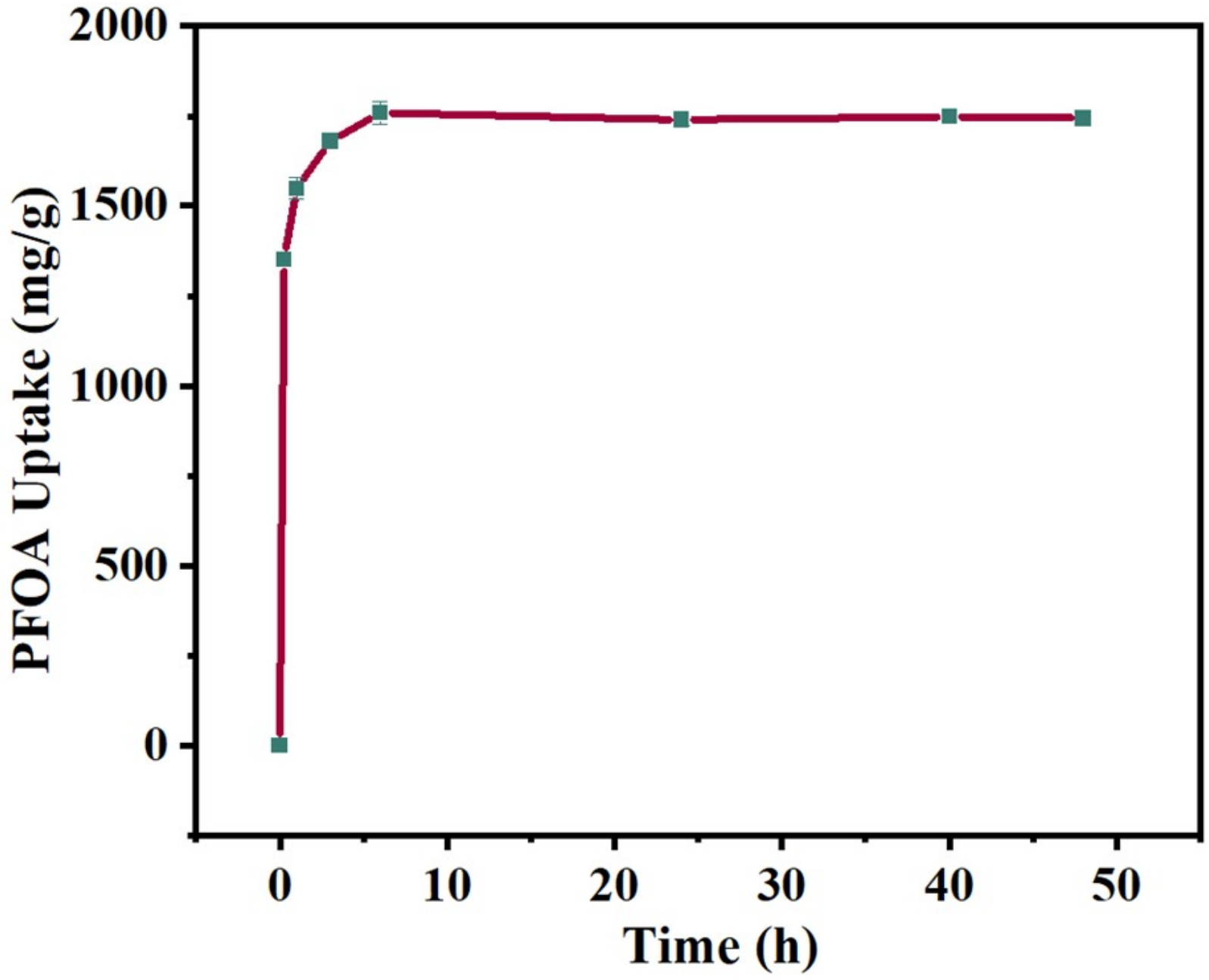

Figure S5. PFOA uptake by HOF-21m (5 mg) from an initial 2000 mg/L (5 mL) PFOA solution. The error bars are the standard deviations calculated from measurements of three independent samples studied in parallel experiments.

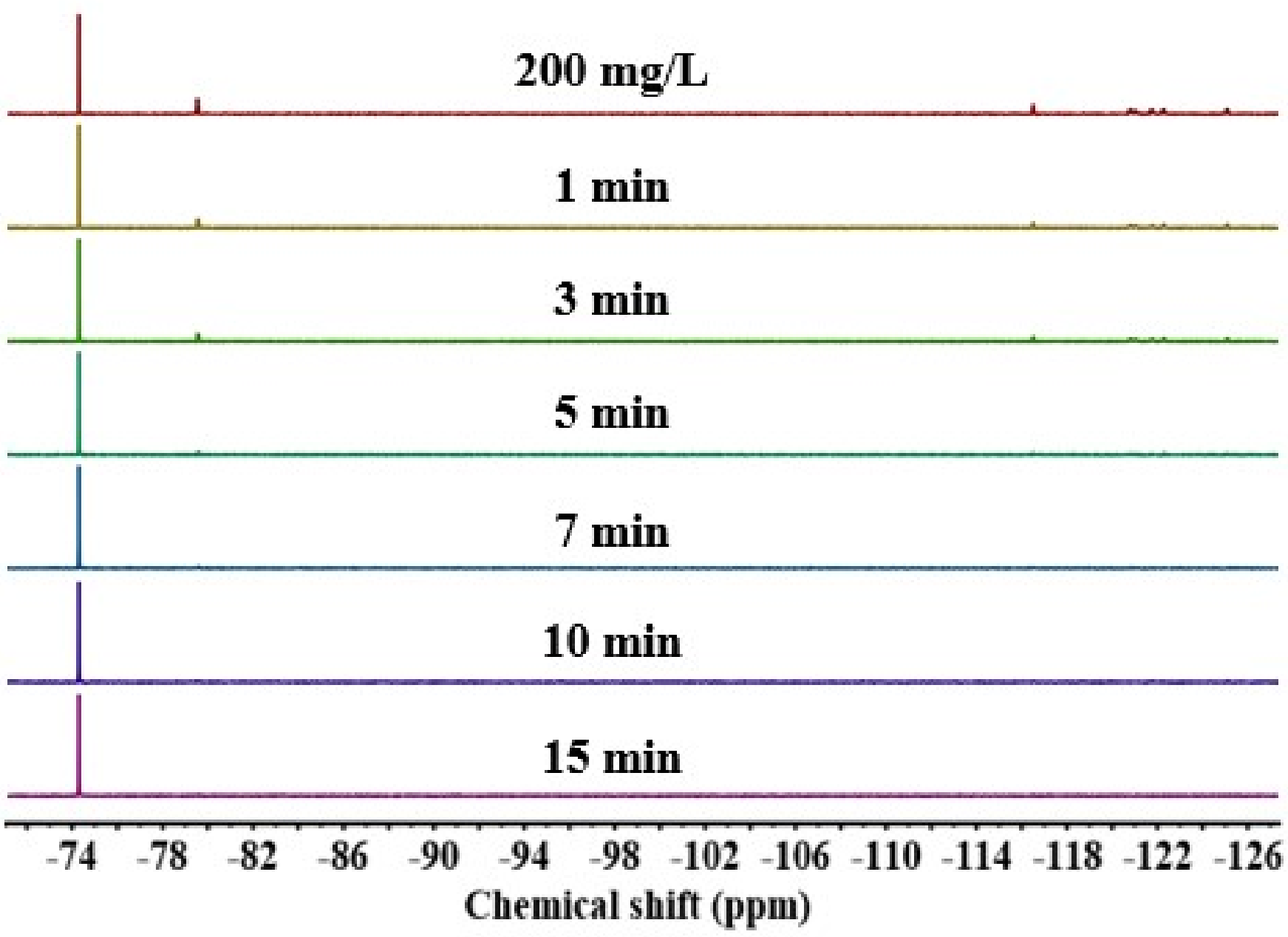

Figure S6. ^{19}F NMR spectra (396 MHz, 298K, H_2O:D_2O = 9:1) of a PFOA solution with an initial concentration of 200 mg/L (5 mL) treated with HOF-21m (5 mg) recorded at different times. Trifluoroacetic acid was used as an internal standard.

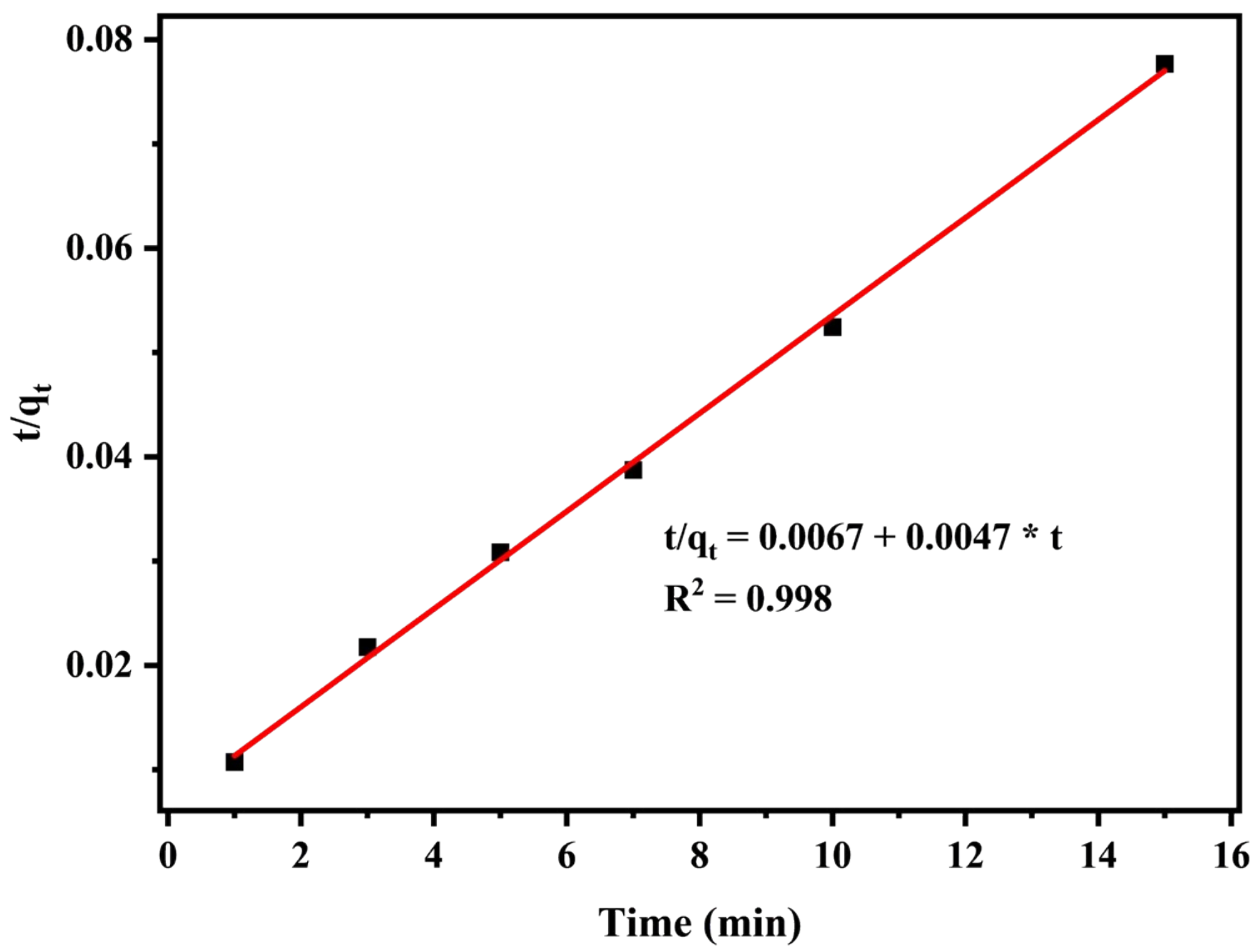

Figure S7. Adsorption kinetics corresponding to the uptake of PFOA with an initial concentration of 200 mg/L (5 mL) by HOF-21m (5 mg).

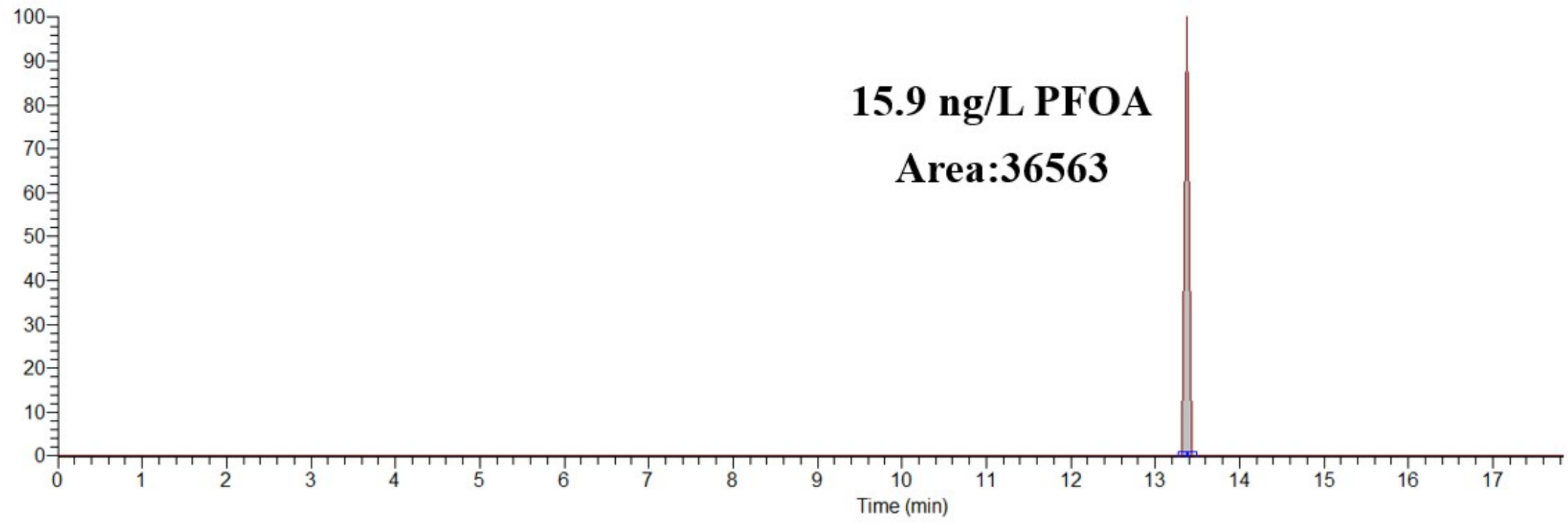

Figure S8. Chromatogram spectra of a PFOA solution with an initial concentration of 200 mg/L (5 mL) treated with HOF-21m powder (5 mg) for 1 h. Note: The residual PFOA concentration remains above the current drinking water guideline level (4 ng/L), highlighting opportunities for further improvement in trace-level PFOA removal.

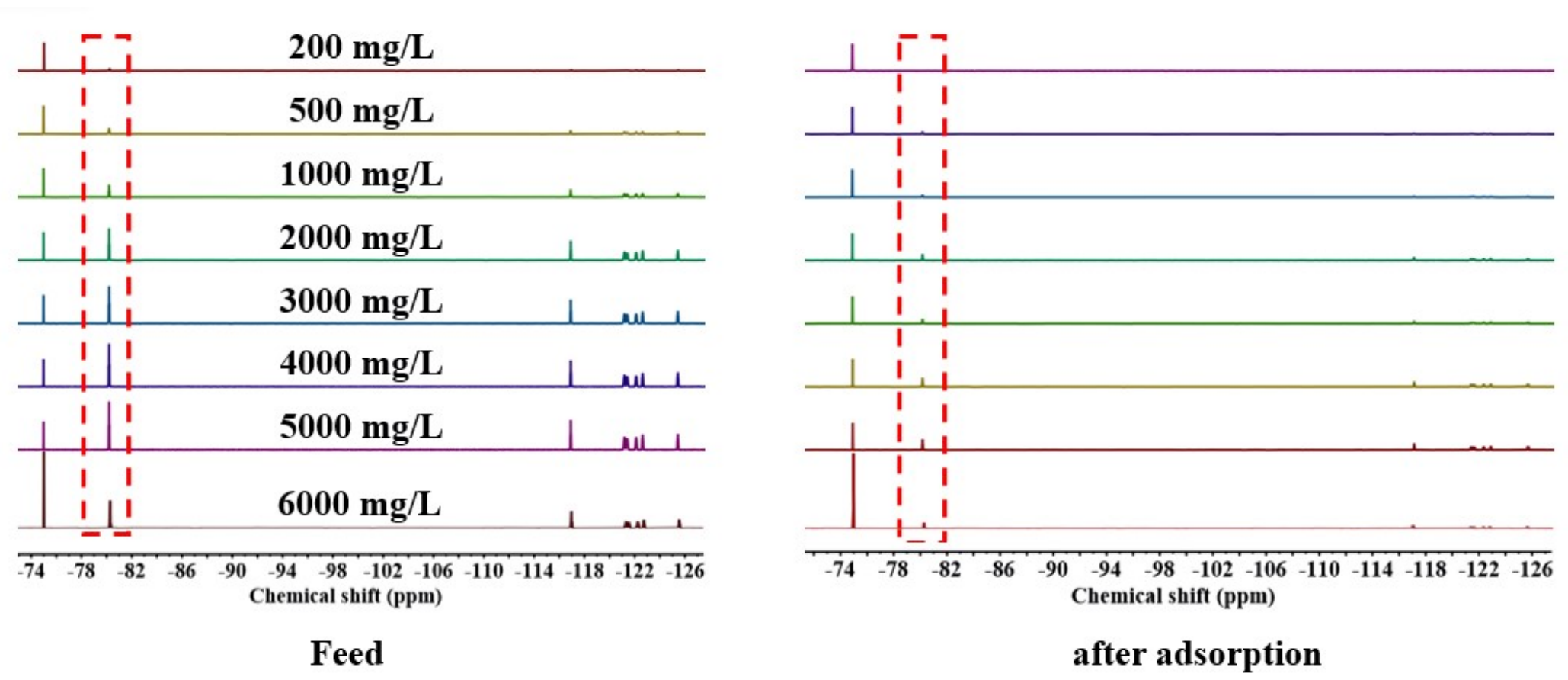

Figure S9. ^{19}F NMR spectra (396 MHz, 298K, H_2O:D_2O = 9:1,v/v) of stock PFOA solutions with initial concentrations ranging from 200 to 5000 mg/L (5 mL) before and after adsorption by HOF-21m (5 mg). Trifluoroacetic acid was used as an internal standard.

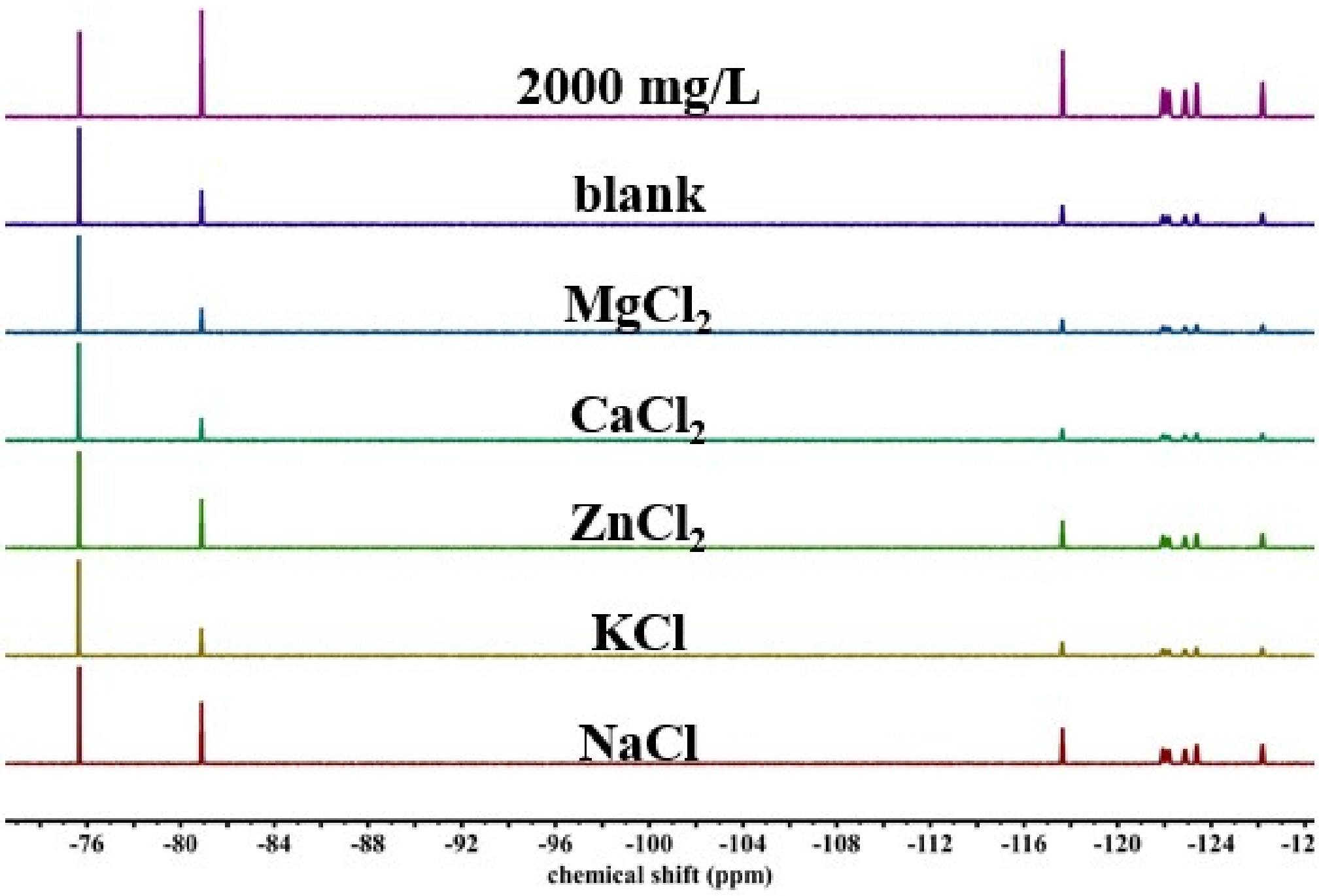

Figure S10. ^{19}F NMR spectra (396 MHz, 298K, H_2O:D_2O = 9:1) of PFOA solutions with an initial concentration of 2000 mg/L (5 mL) containing added salts and treated with HOF-21m (5 mg). Trifluoroacetic acid was used as an internal standard.

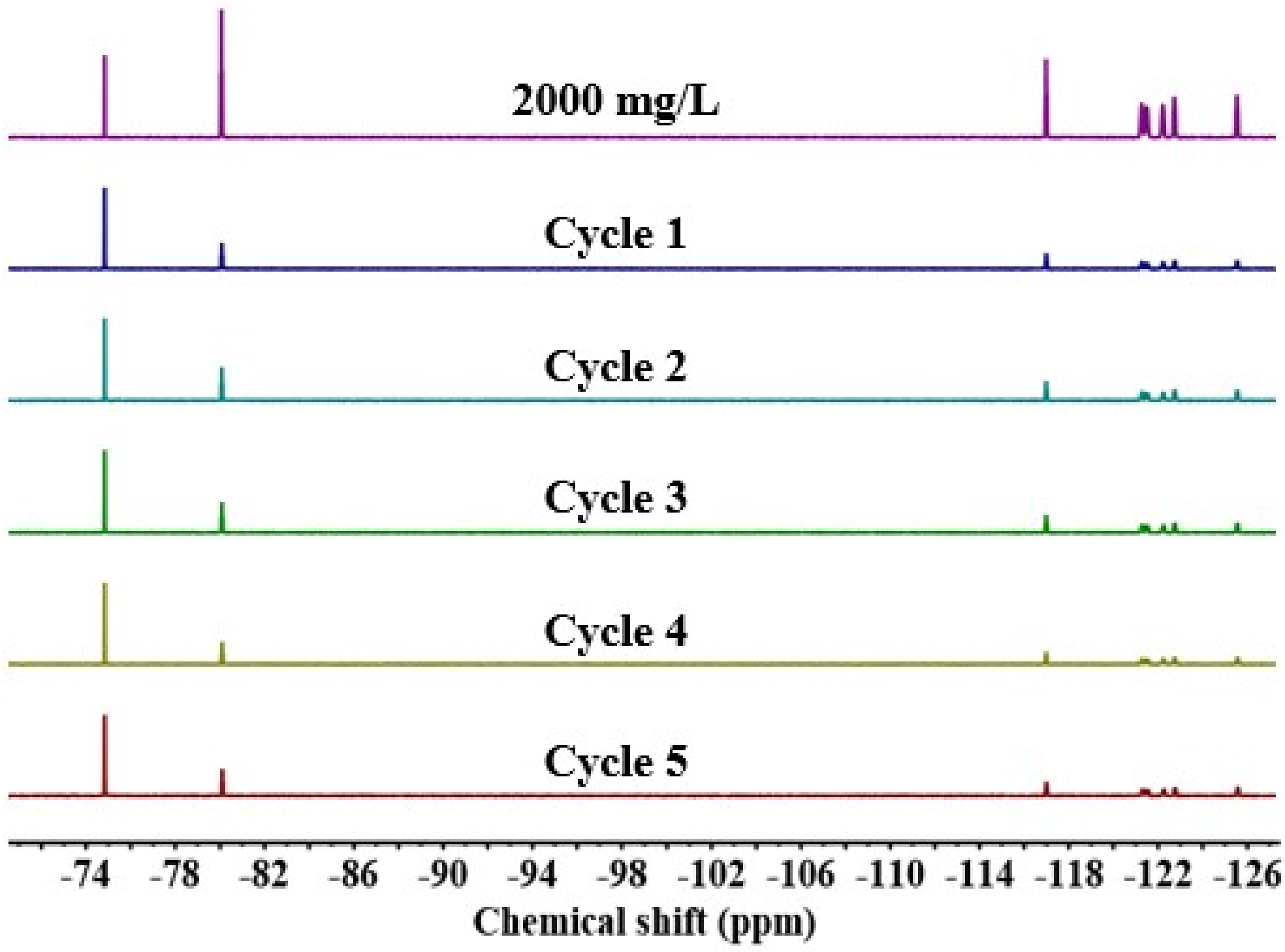

Figure S11. ^{19}F NMR spectra (396 MHz, 298K, $H_2O:D_2O = 9:1$) of PFOA solutions with an initial concentration of 2000 mg/L (5 mL) treated with HOF-21m (5 mg) through different regeneration cycles. Trifluoroacetic acid was used as an internal standard.

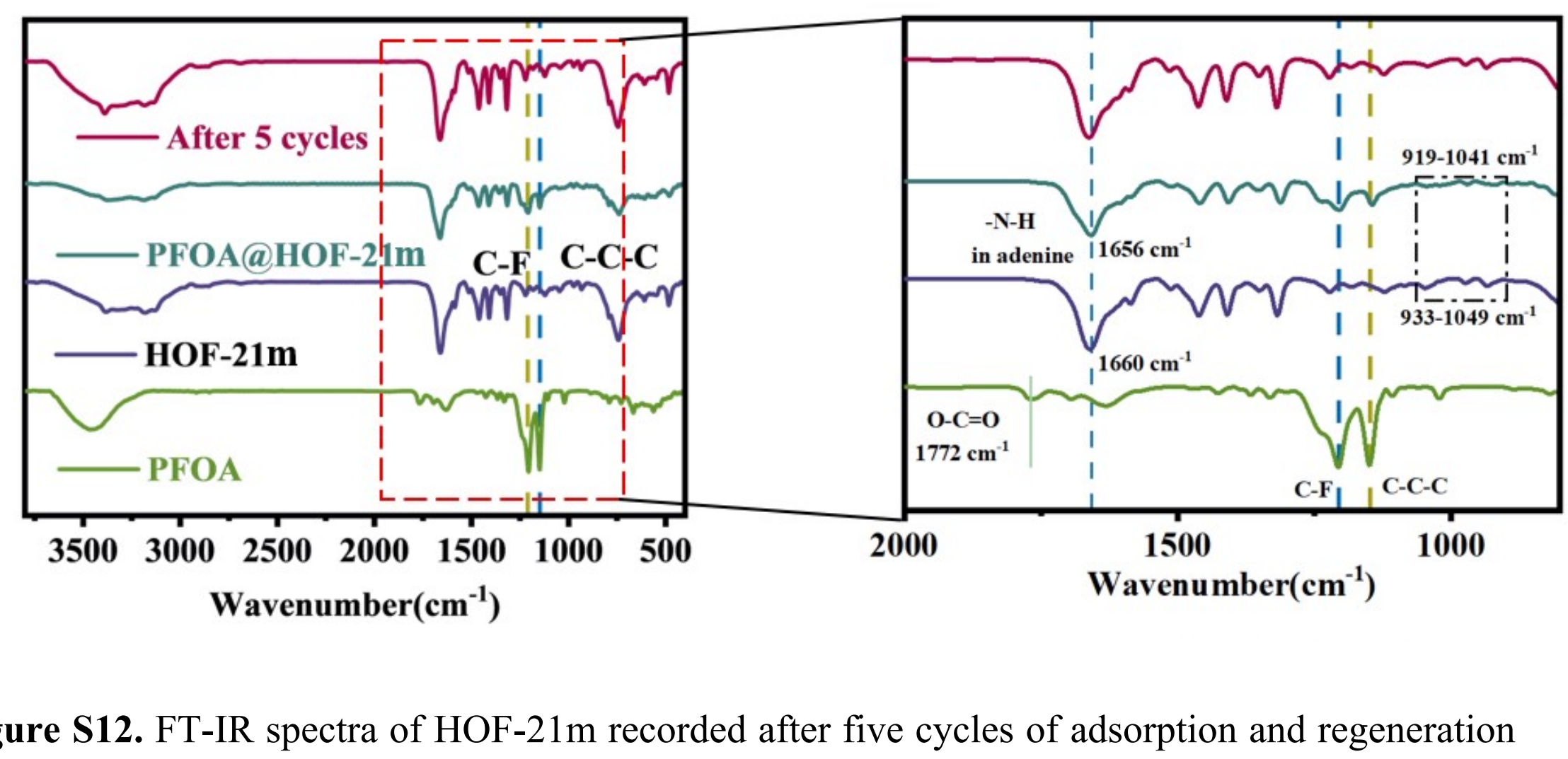

Figure S12. FT-IR spectra of HOF-21m recorded after five cycles of adsorption and regeneration (red line), PFOA@HOF-21 (bluish-green), before use (purple line) and after contact with PFOA (green line).

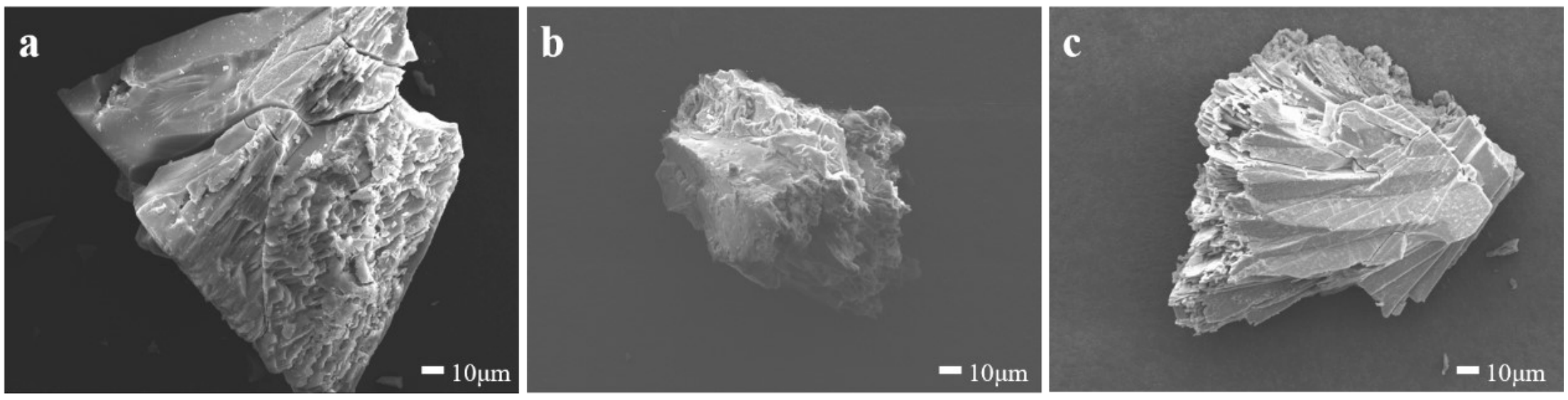

Figure S13. SEM images of (a) as-prepared HOF-21m, (b) HOF-21m after PFOA adsorption, and (c) HOF-21m after five adsorption–regeneration cycles.

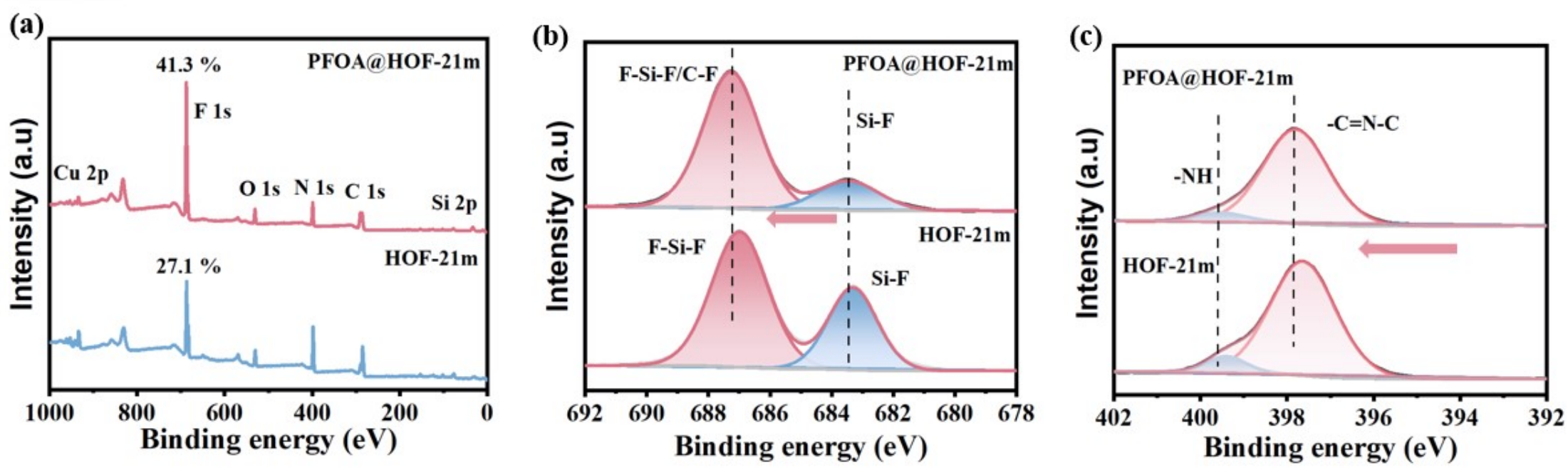

Figure S14. (a) XPS survey spectrum for HOF-21m and PFOA@HOF-21m. (b) High-resolution F 1s analysis of HOF-21m and PFOA@HOF-21m. (c) High-resolution N 1s analysis of HOF-21m and PFOA@HOF-21m.

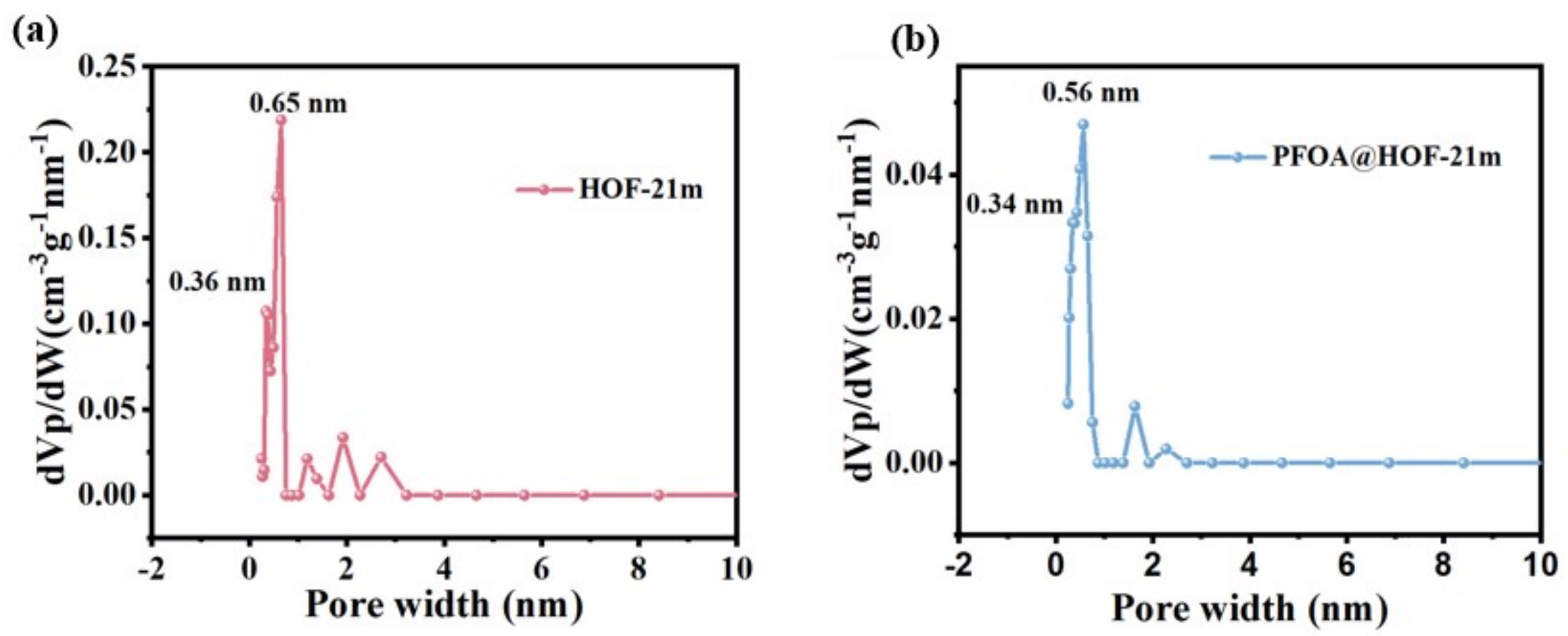

Figure S15. (a) Pore size distribution of HOF-21m. (b) Pore size distribution of PFOA@HOF-21m.

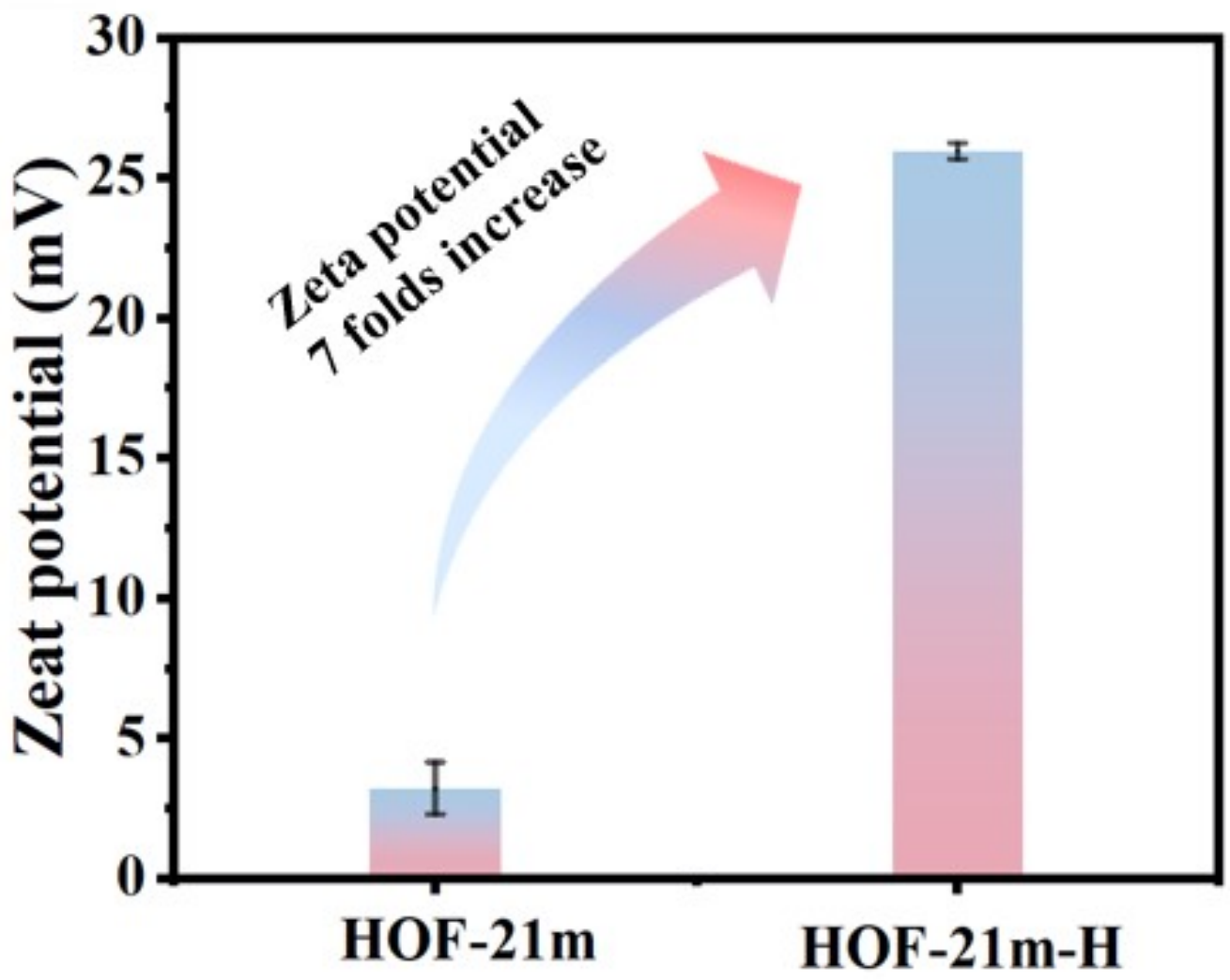

Figure S16. The Zeta potential of HOF-21m and HOF-21m-H.

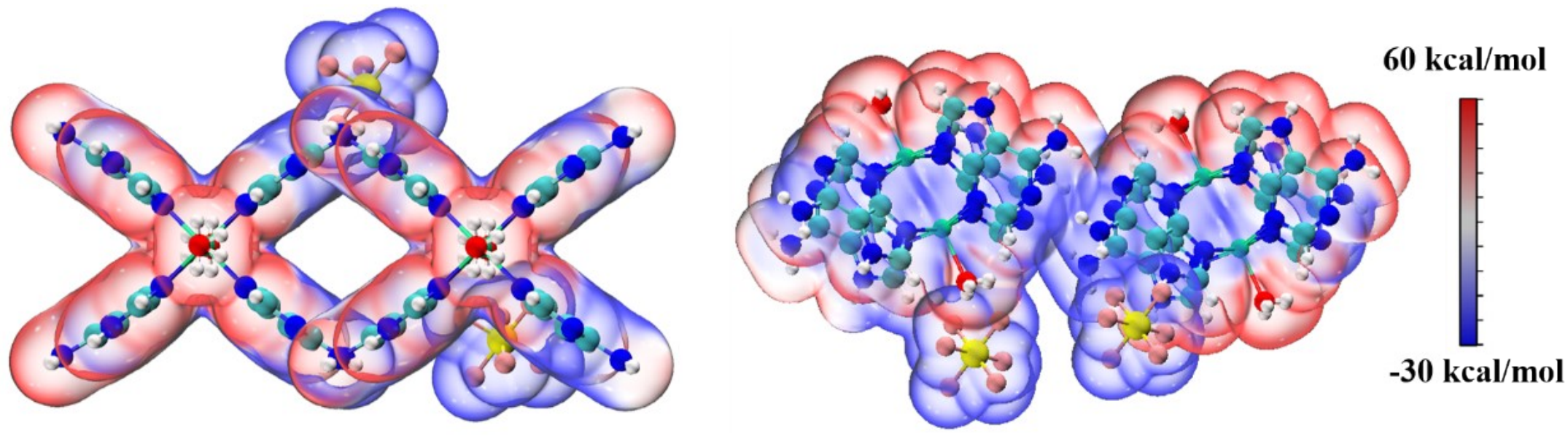

Figure S17. Molecular electrostatic potential (MESP) distribution of a model for HOF-21m seen in different views.

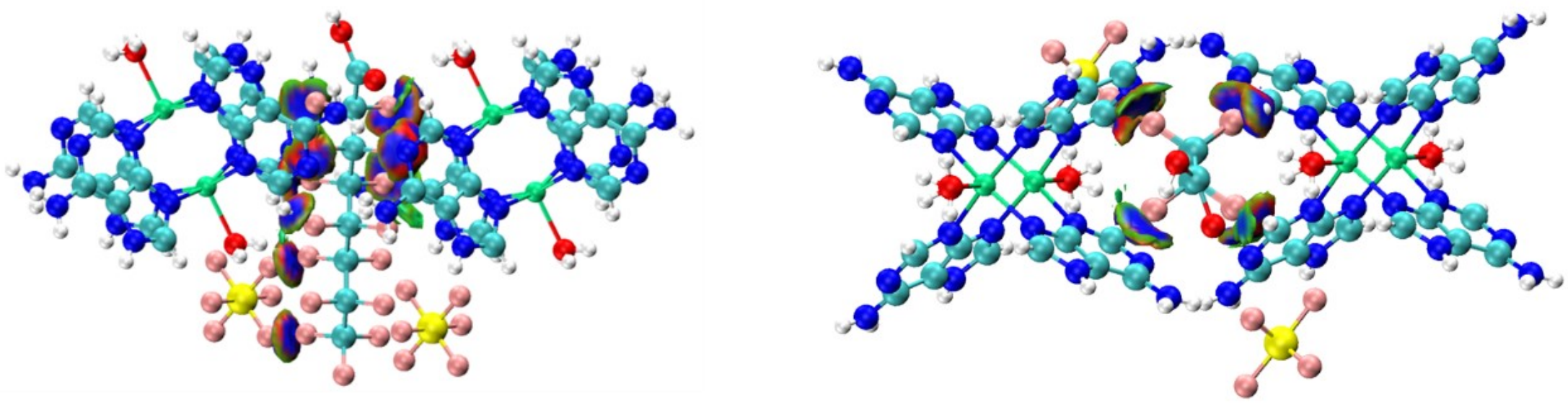

Figure S18. Contour plots of independent gradient model based on Hirshfeld partition (IGMH) isosurfaces for a HOF-21m model compound with PFOA (site 1) as seen in different views. Note: C, H, O, F, Si, and Cu atoms are represented by cyan, white, red, pink, yellow, and green, respectively.

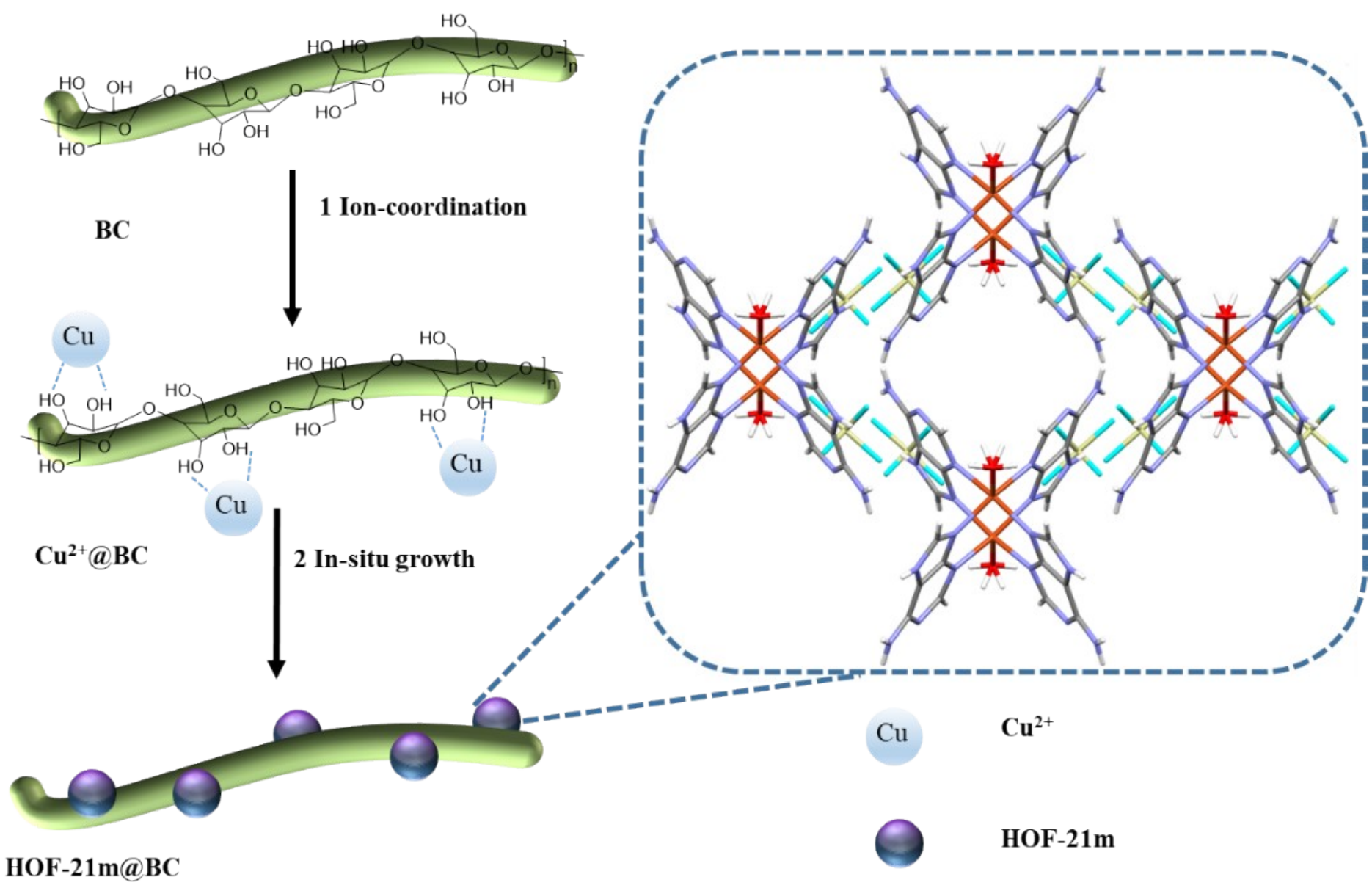

Figure S19. Schematic illustration of the in-situ preparation of the HOF-21m@BC composite membrane.

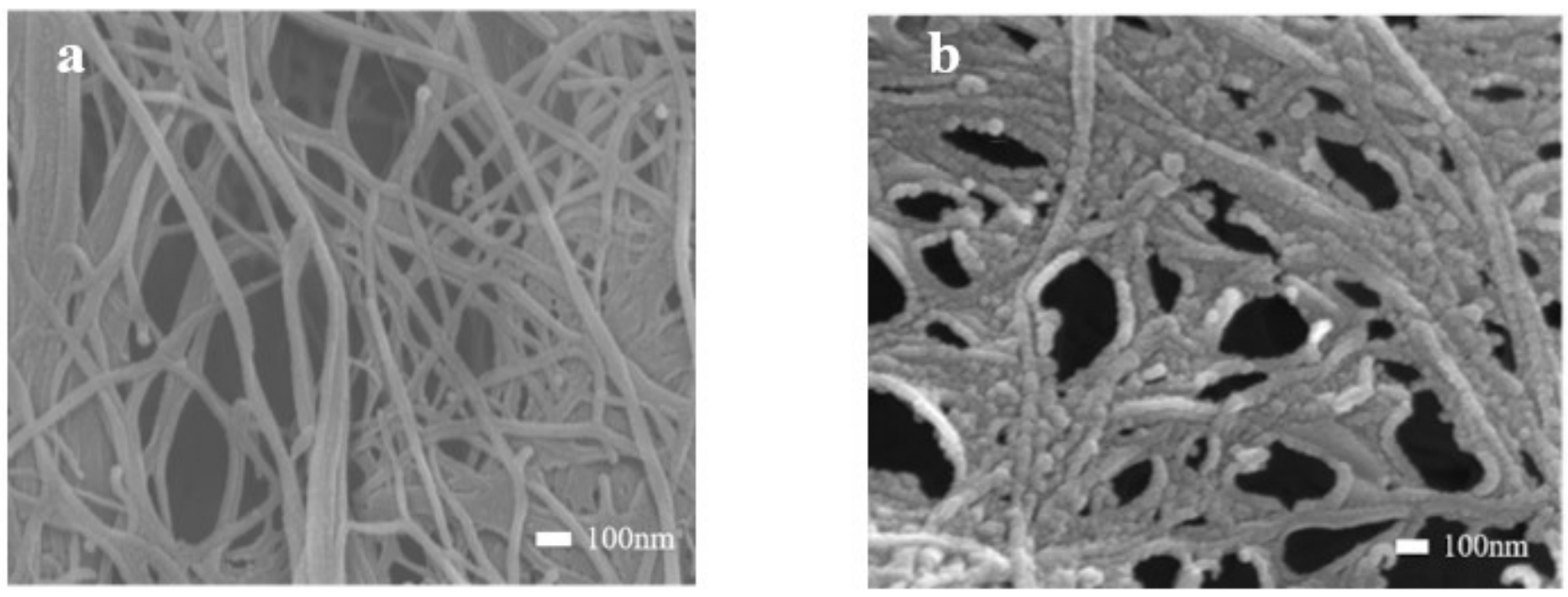

Figure S20. SEM images of (a) pristine bacterial cellulose (BC) and (b) HOF-21m@BC composite membranes.

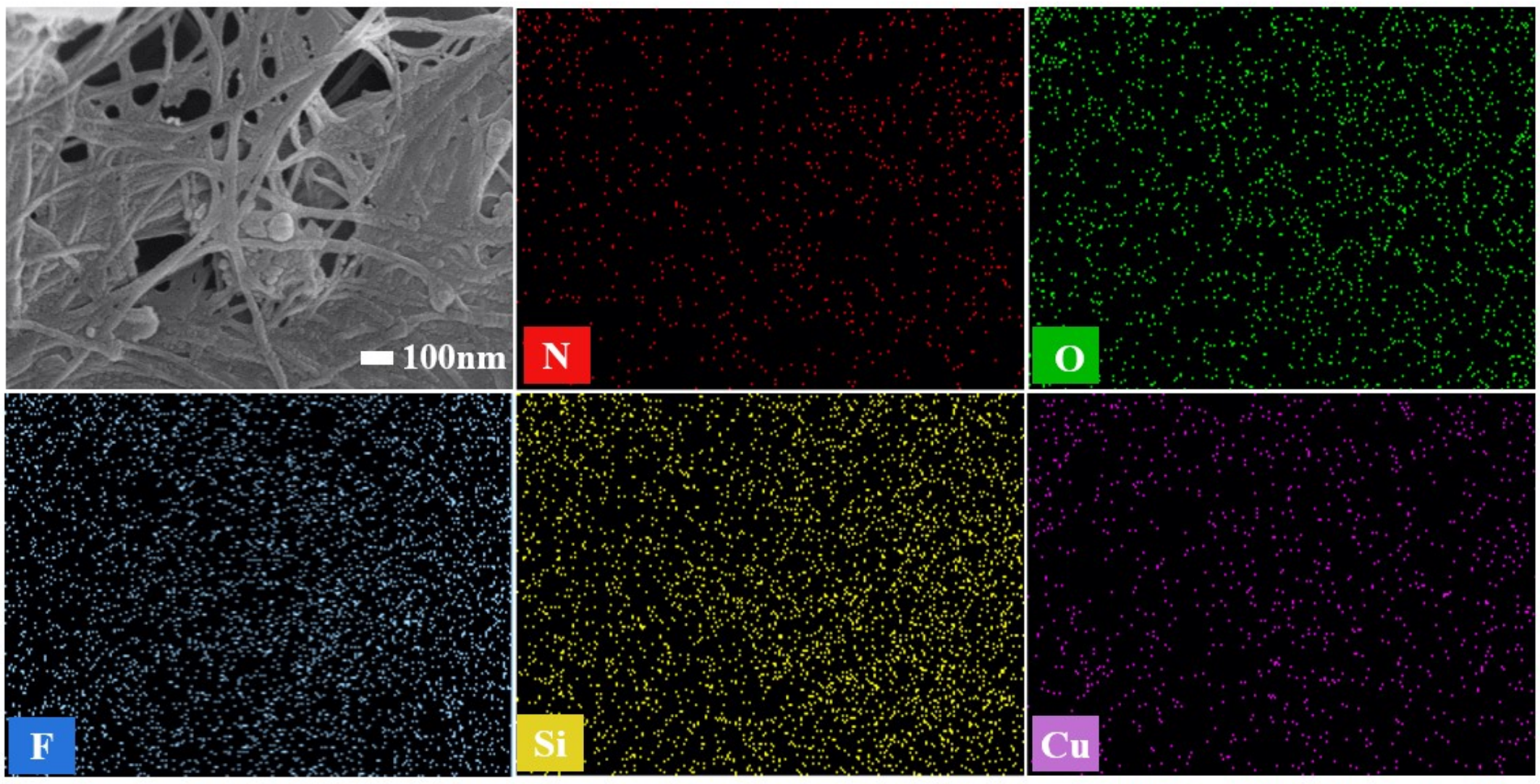

Figure S21. EDX elemental mapping images of the HOF-21m@BC composite membrane. The uniform distribution of key elements (F, Si, and Cu) across the membrane is taken as evidence for the successful incorporation of HOF-21m onto the cellulose substrate.

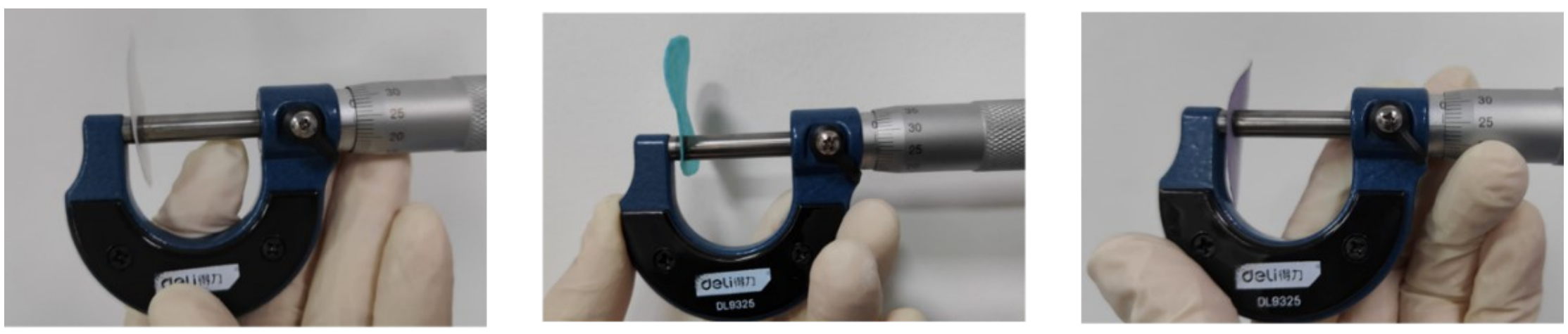

BC **Cu^{2+}@BC** **HOF-21@BC**

Figure S22. Measurements of the thickness of pristine BC (229 μm), Cu^{2+}@BC (265 μm), and HOF-21m@BC (241 μm).

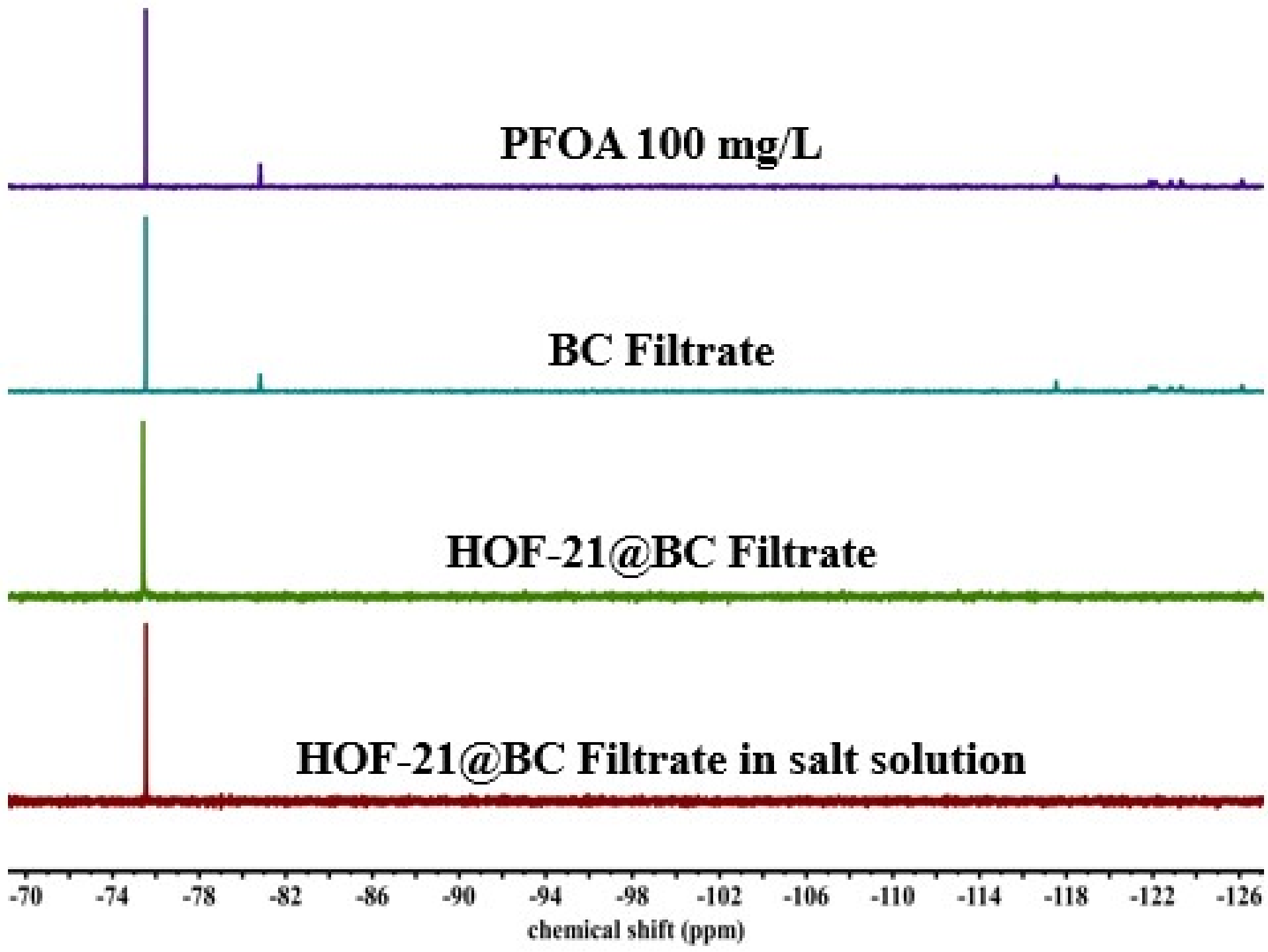

Figure S23. ^{19}F NMR spectra (396 MHz, 298K, H_2O:D_2O = 9:1) of PFOA solutions filtered through BC, HOF-21m@BC, and HOF-21m@BC in the presence of mixed salts (100 mg/L PFOA; 0.05 mM each of $MgCl_2$, $CaCl_2$, $ZnCl_2$, KCl, and NaCl). These spectral data are taken as evidence for the superior PFOA removal capability of HOF-21m@BC and its tolerance to ionic interference.

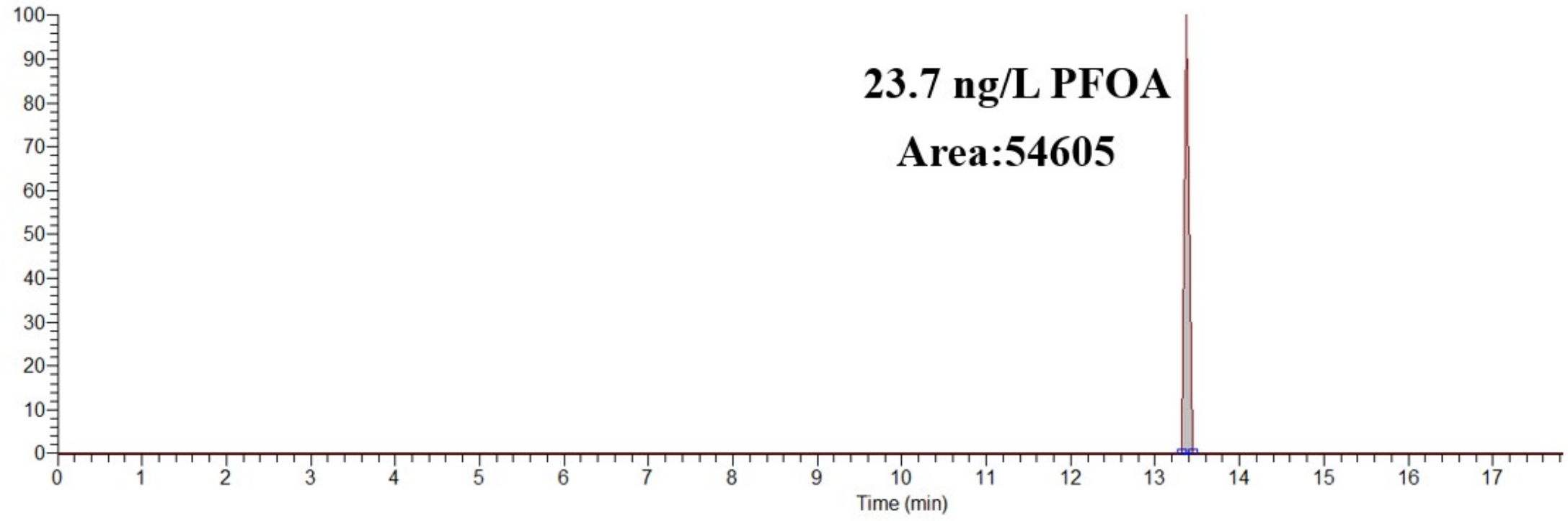

Figure S24. Chromatogram of a PFOA solution (initial concentration: 100 mg/L) after treatment with the HOF-21m@BC composite membrane. Note: The residual PFOA concentration remains above the current drinking water guideline level (4 ng/L), indicating opportunities for further improvement in trace-level PFOA removal.

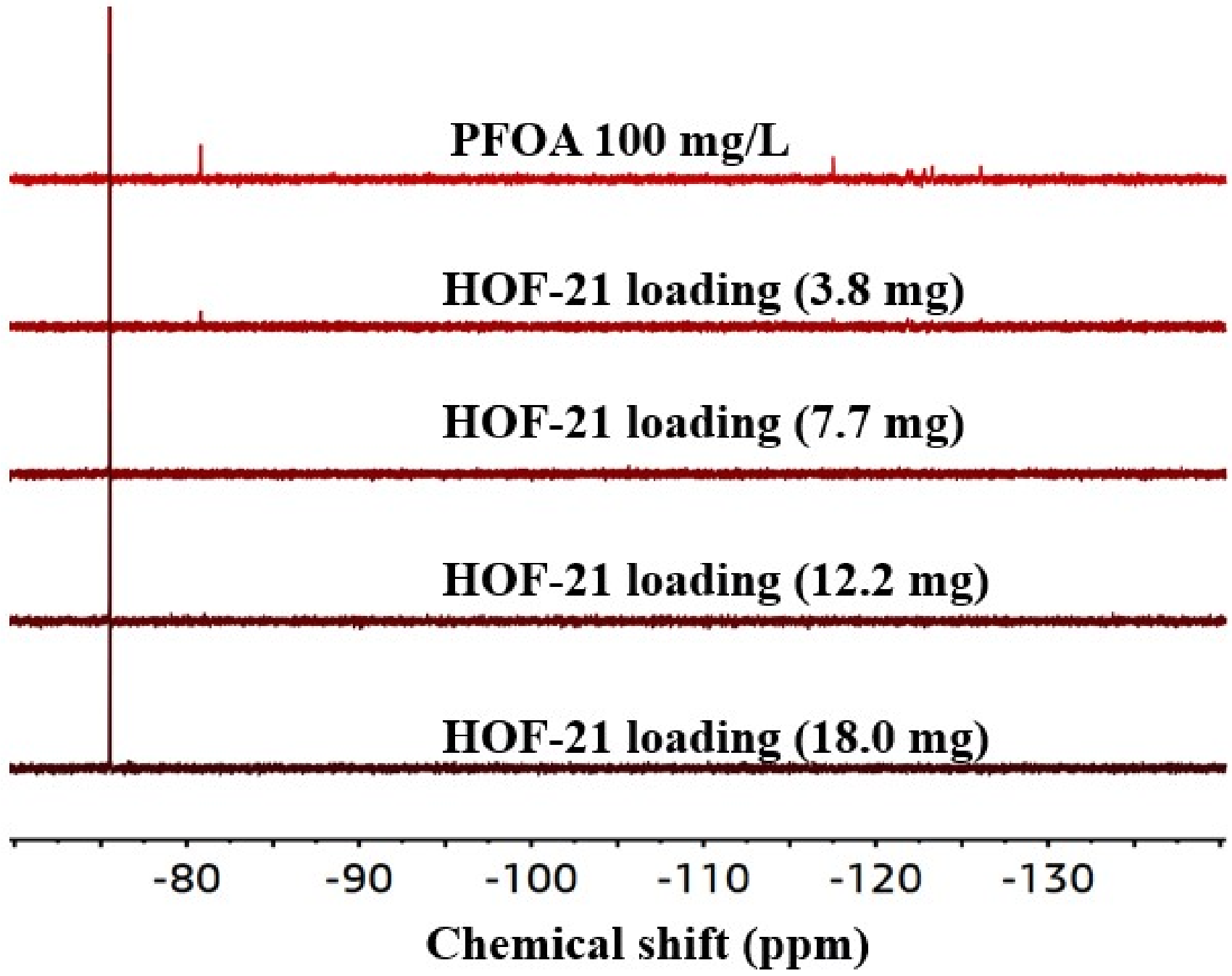

Figure S25. ^{19}F NMR spectra (396 MHz, 298K, $H_2O:D_2O$ = 9:1, v/v) of PFOA solutions after being filtered through HOF-21m@BC membranes under different HOF-21 loadings.

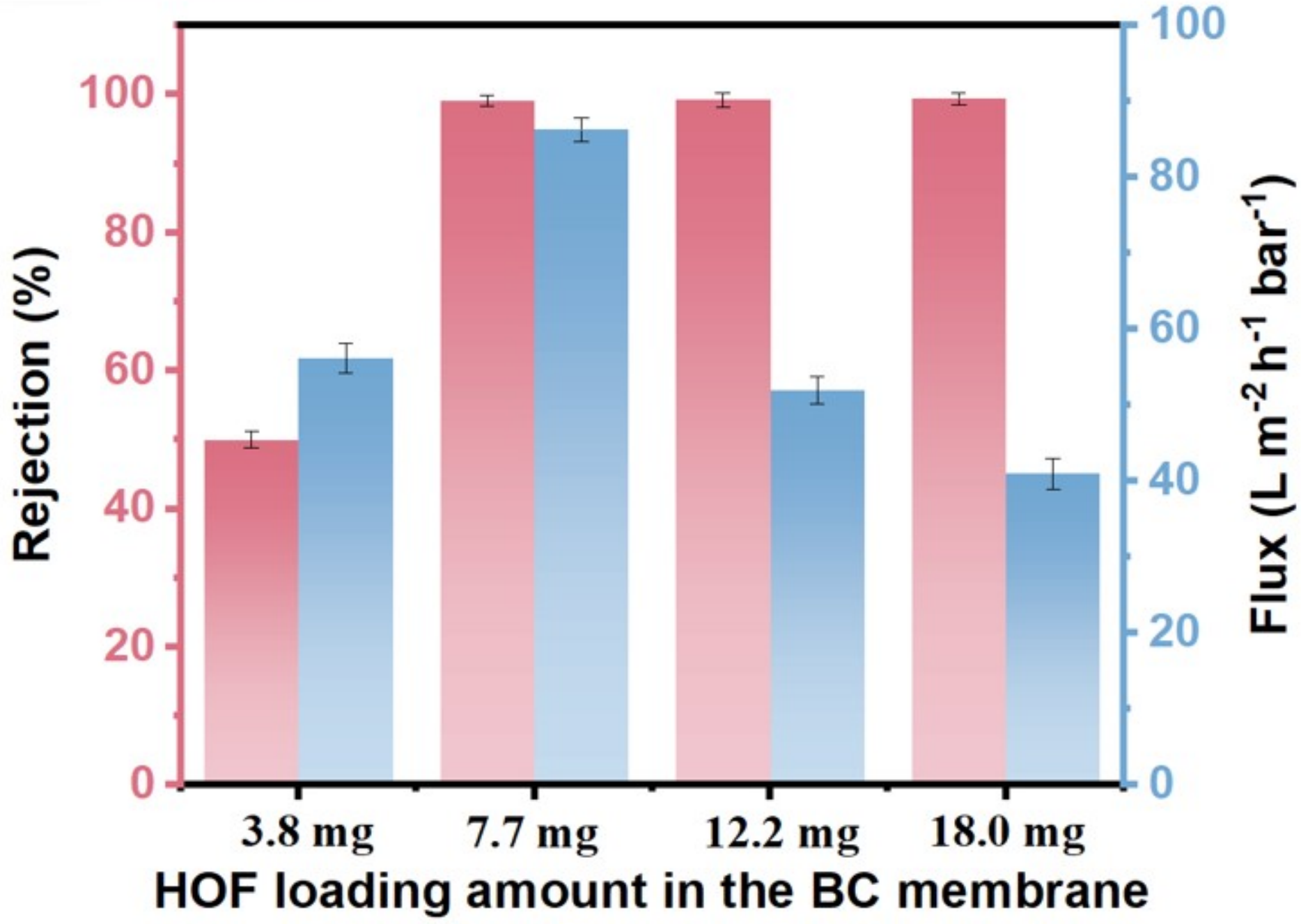

Figure S26. PFOA rejection efficiency and water permeance of HOF-21m@BC membranes as a function of HOF-21m loading.

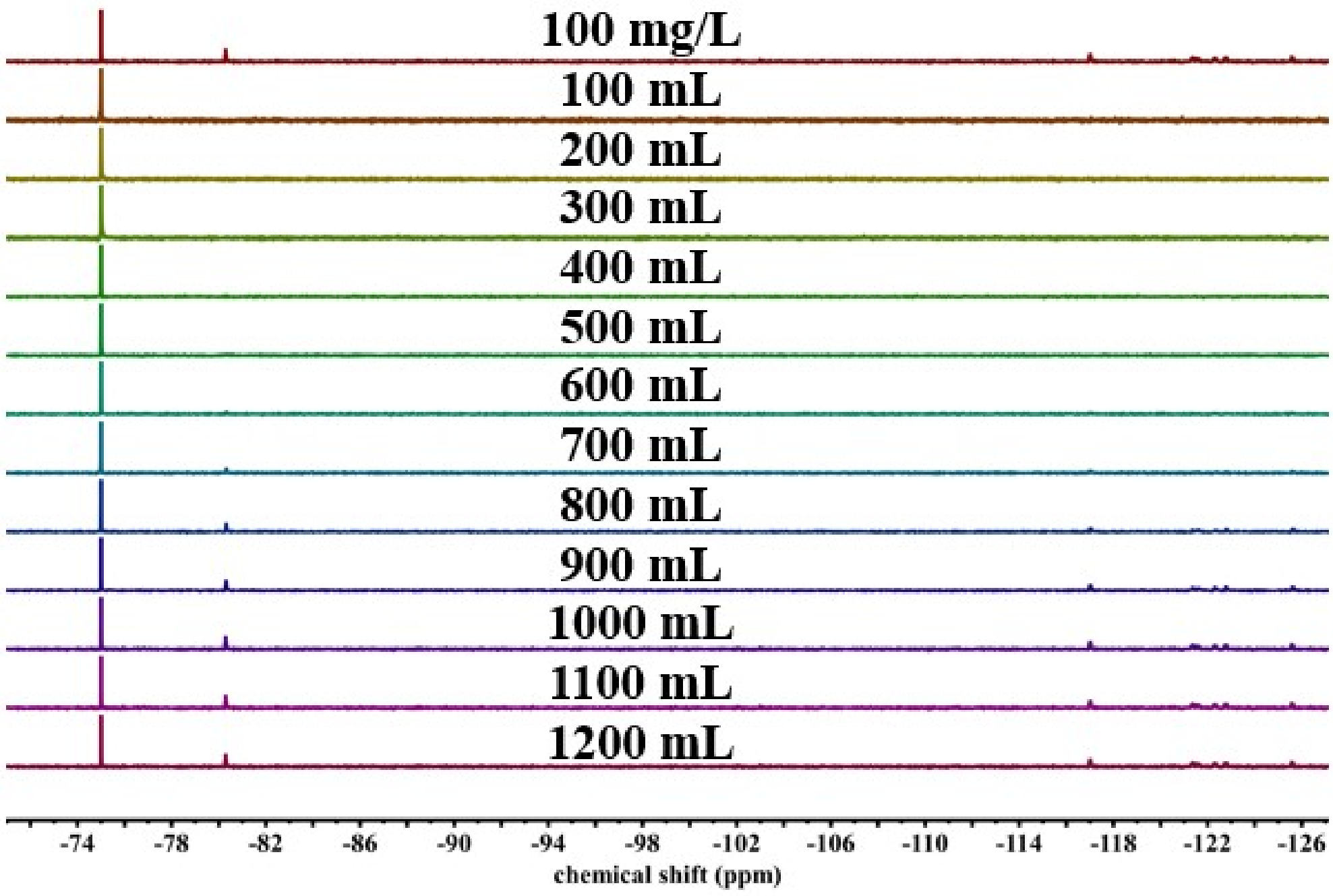

Figure S27. ^{19}F NMR (396 MHz, 298K, $H_2O:D_2O=9:1$) spectra of PFOA solution filtrated through HOF-21m@BC after breakthrough. Trifluoroacetic acid was used as an internal standard.

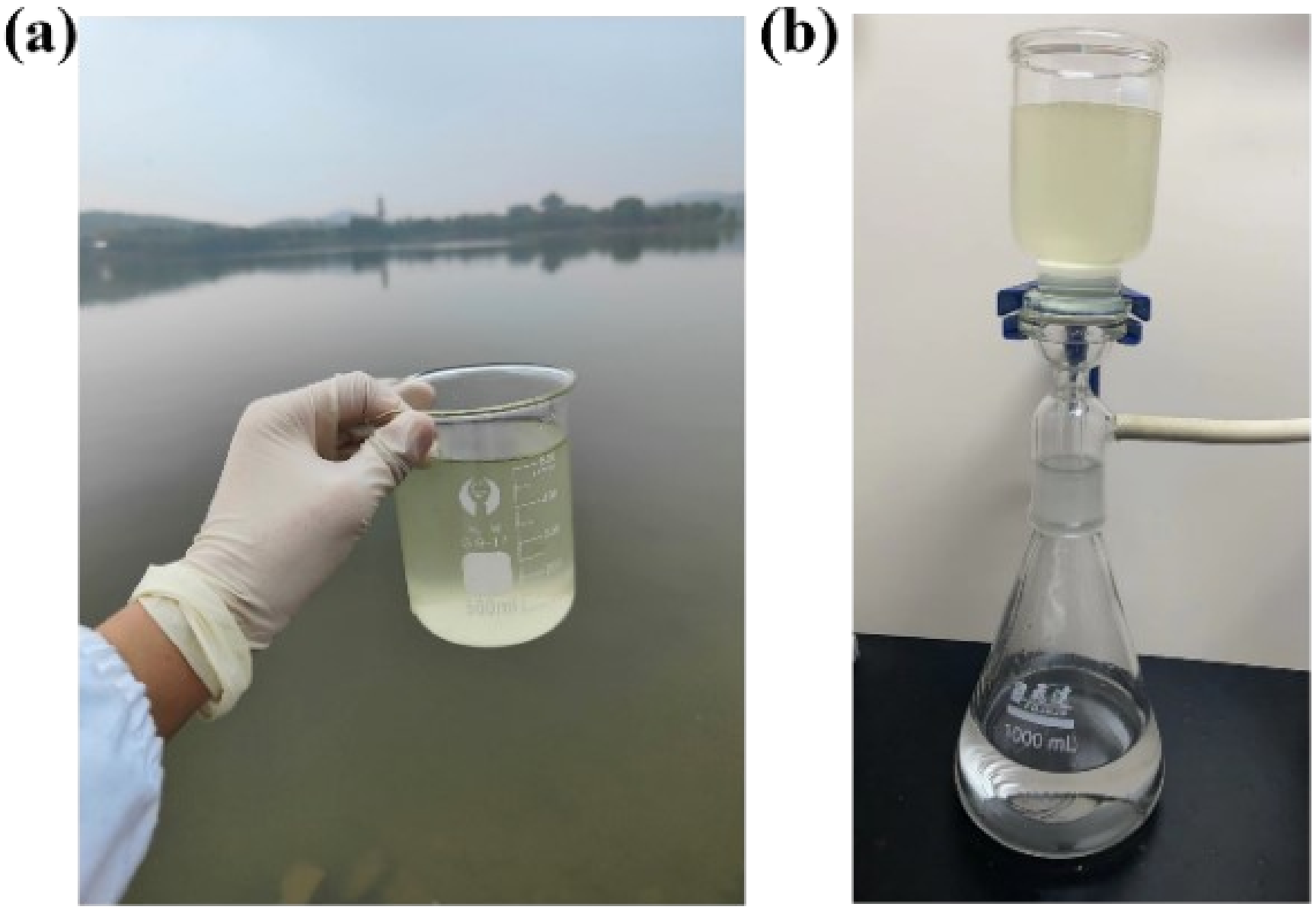

Figure S28. (a) Photograph showing the collection of a lake water sample. (b) Preparation of a 100 ppm PFOA solution using East Lake water. Note: Because the actual PFOA concentration in East Lake water was extremely low (84.9 ng/L), additional PFOA was spiked into the lake water to achieve a concentration of 100 mg/L.

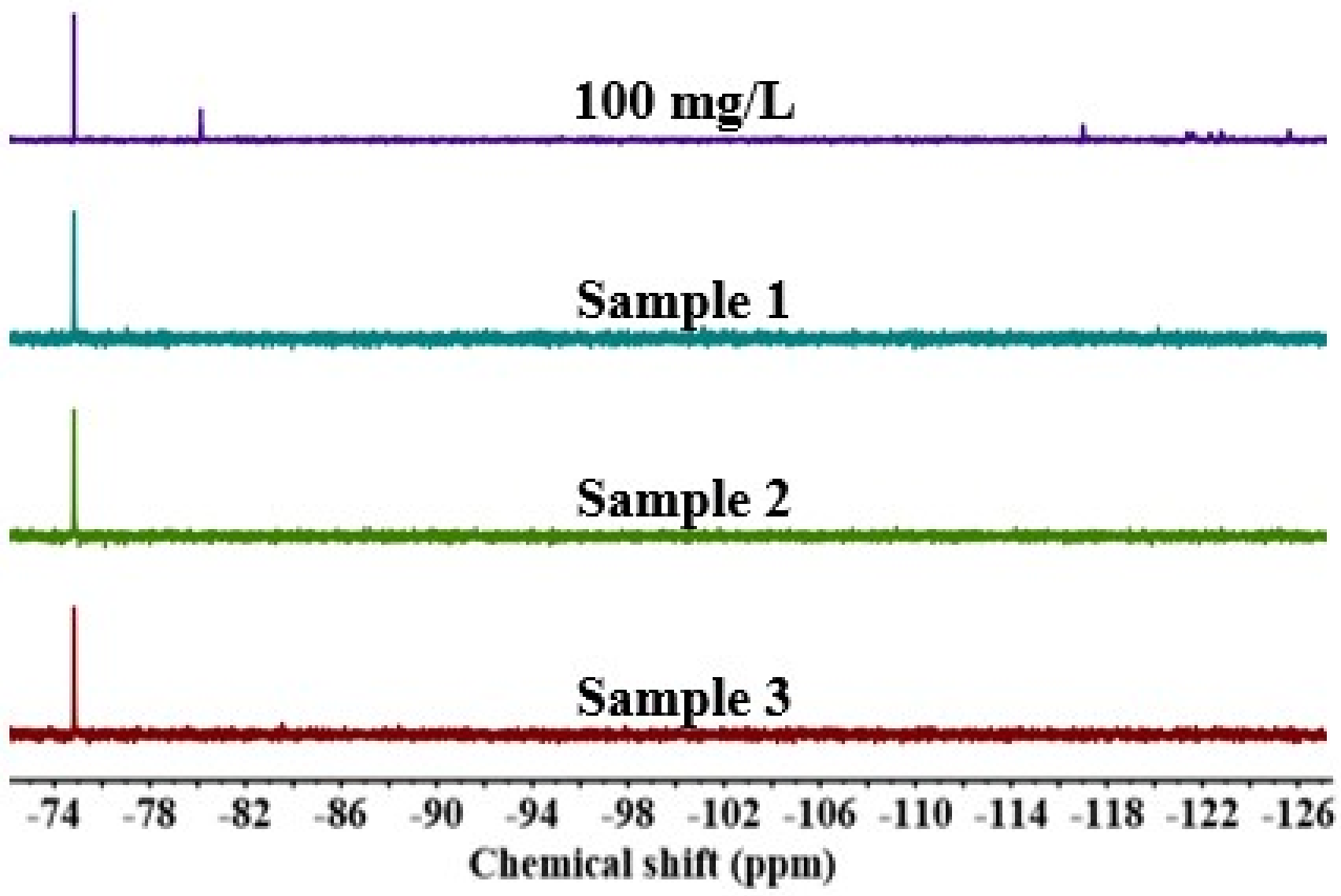

Figure S29. ^{19}F NMR spectra (396 MHz, 298K, H_2O:D_2O=9:1) of PFOA solutions filtrated through HOF-21m@BC. Trifluoroacetic acid was used as an internal standard.

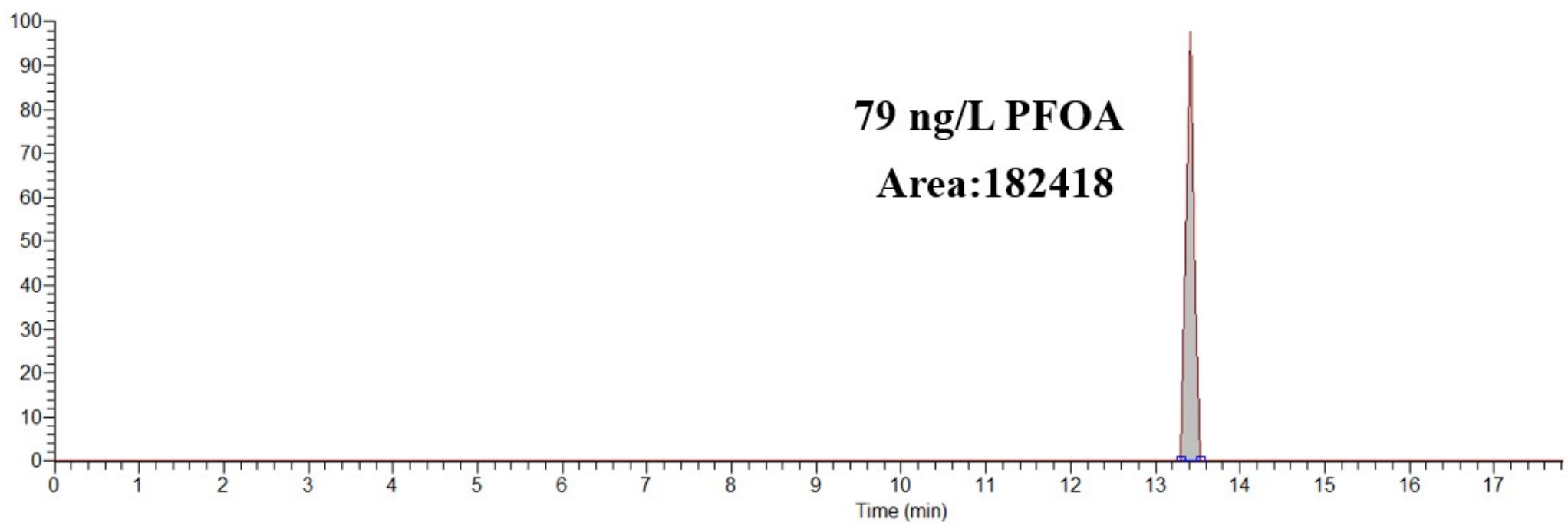

Figure S30. Chromatogram of a PFOA-spiked lake water solution (initial concentration: 100 mg/L) after treatment with the HOF-21m@BC composite membrane. Note: The residual PFOA concentration remains above the current drinking water guideline level (4 ng/L), indicating opportunities for further improvement in trace-level PFOA removal.

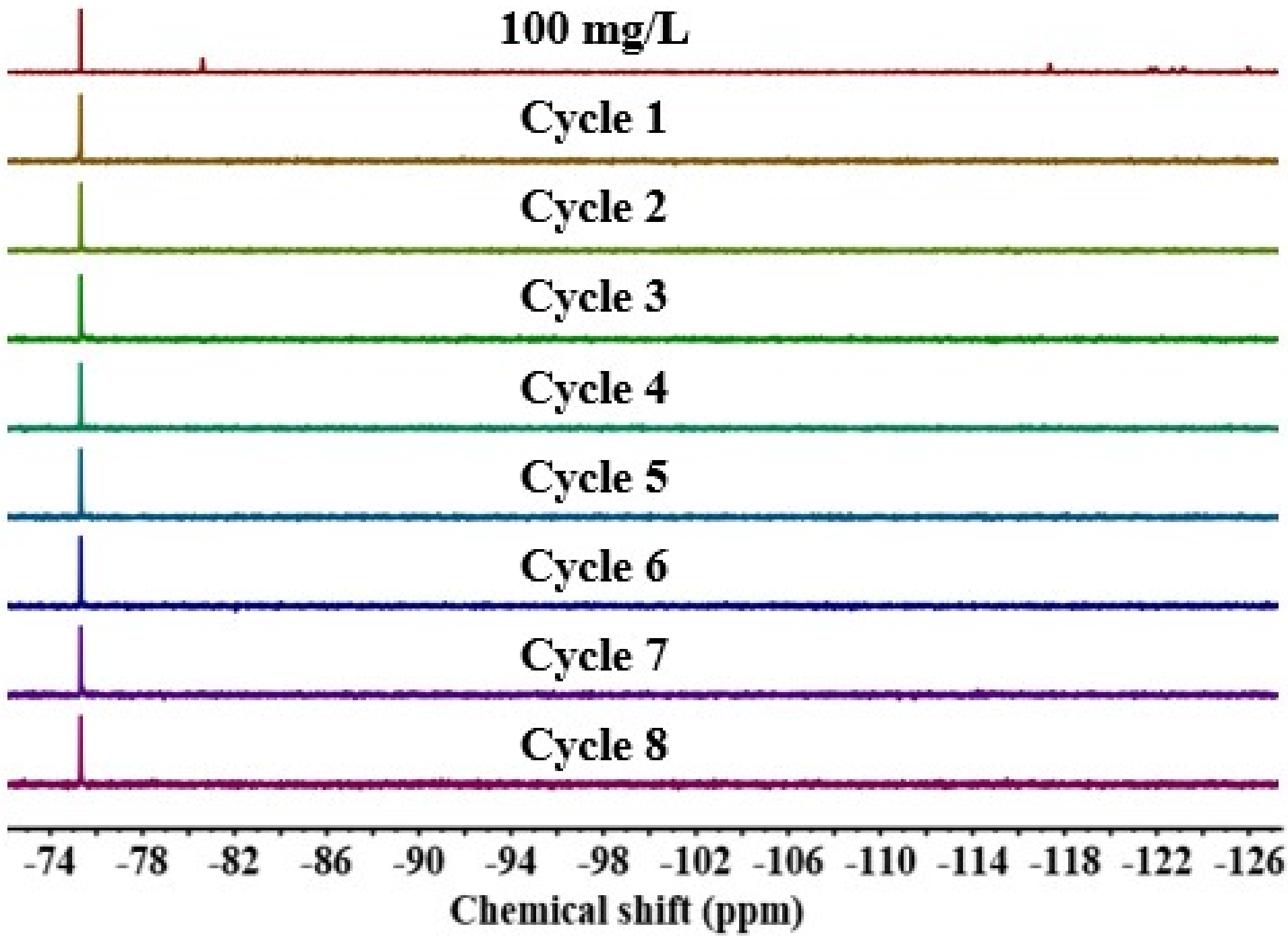

Figure S31. ^{19}F NMR spectra (396 MHz, 298K, $H_2O:D_2O$ = 9:1) of a PFOA solution filtrated through HOF-21m@BC across different regeneration cycles, showing the retained adsorption efficiency of the membrane. Trifluoroacetic acid was used as an internal standard.

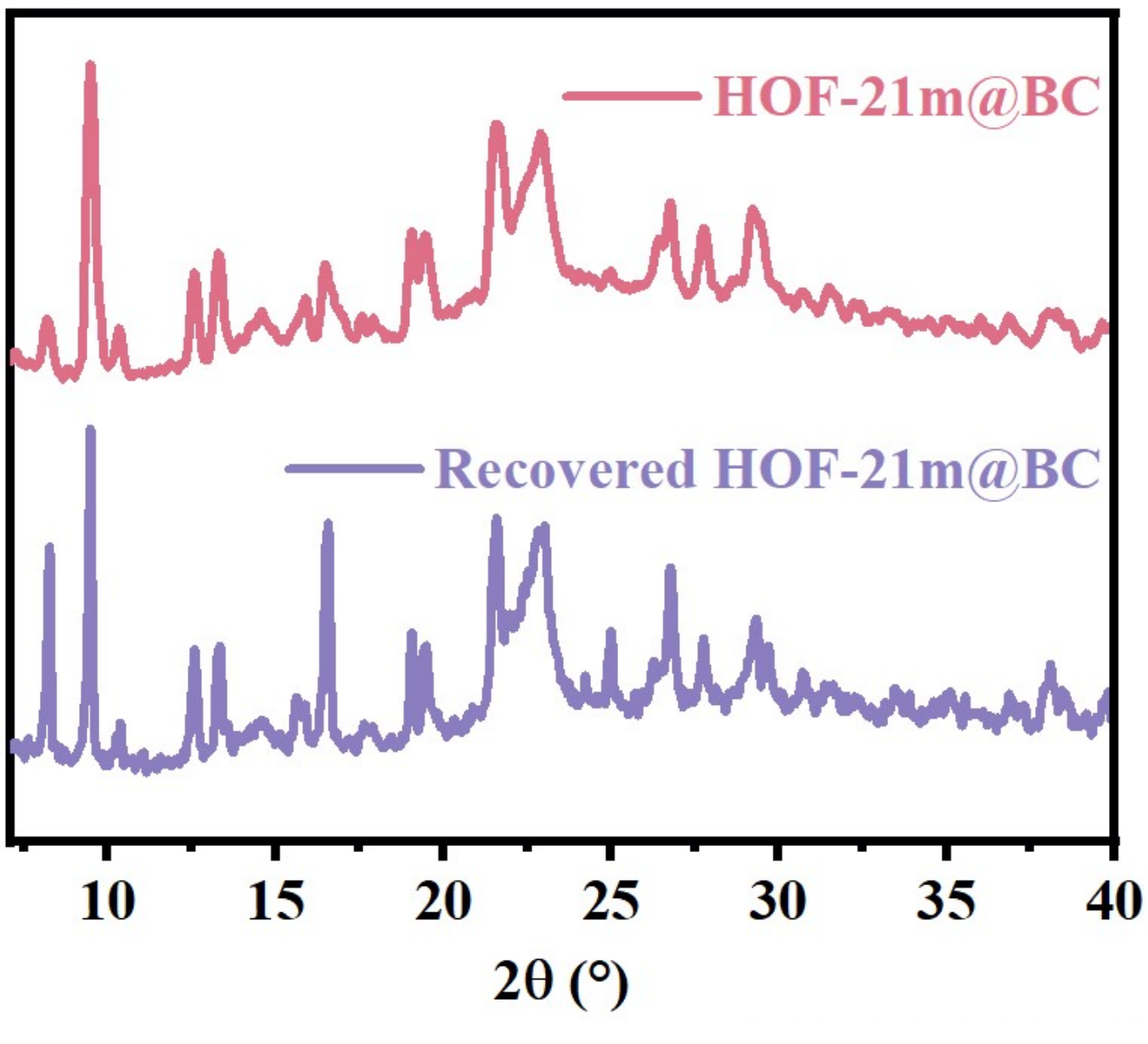

Figure S32. PXRD patterns of as-prepared and recovered HOF-21m@BC membranes. The similarity of the patterns indicates that the crystalline structure of HOF-21m is largely retained after use, demonstrating the stability of the composite membrane.

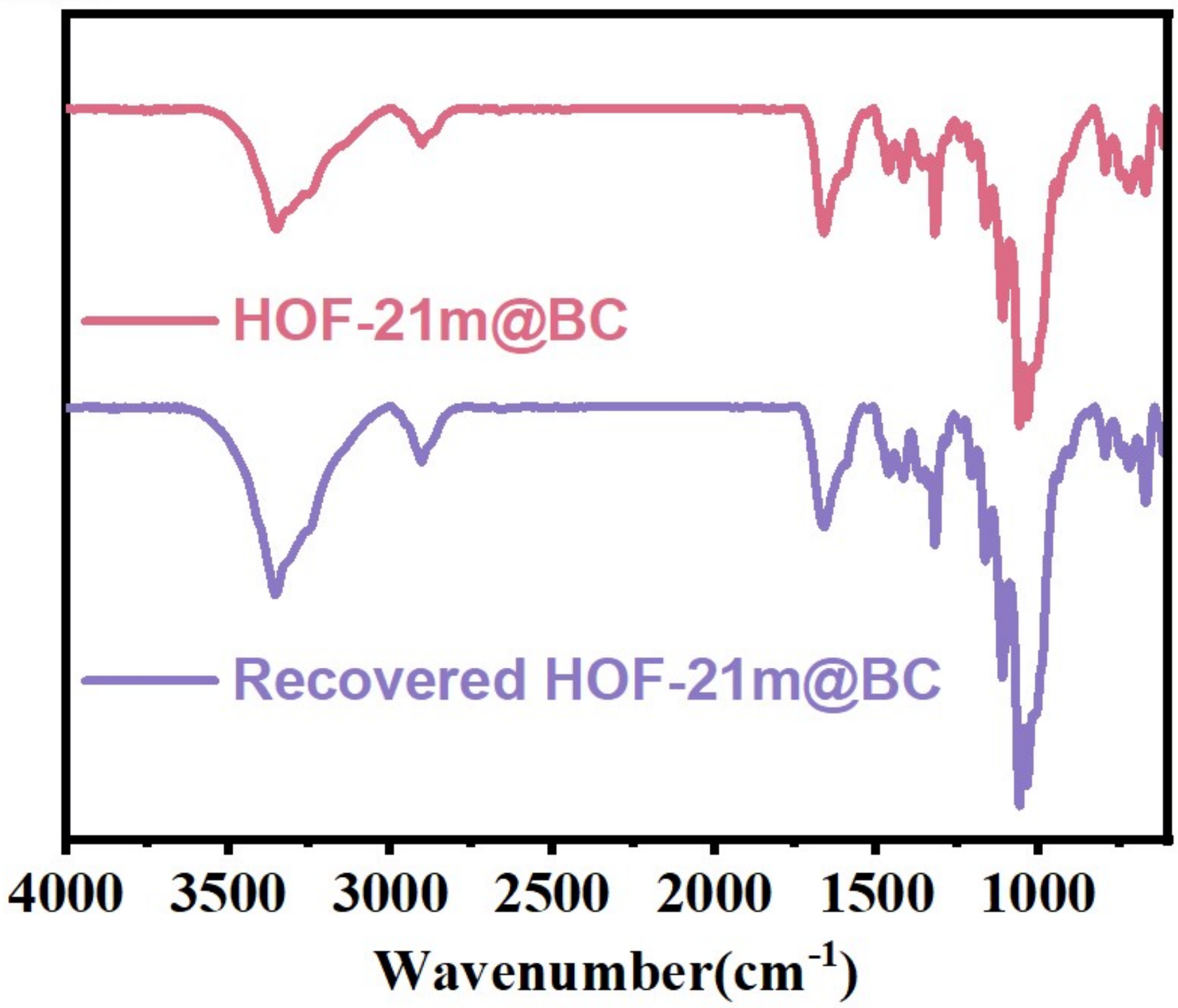

Figure S33. FT-IR spectra of recovered HOF-21m@BC and as-prepared HOF-21m@BC.

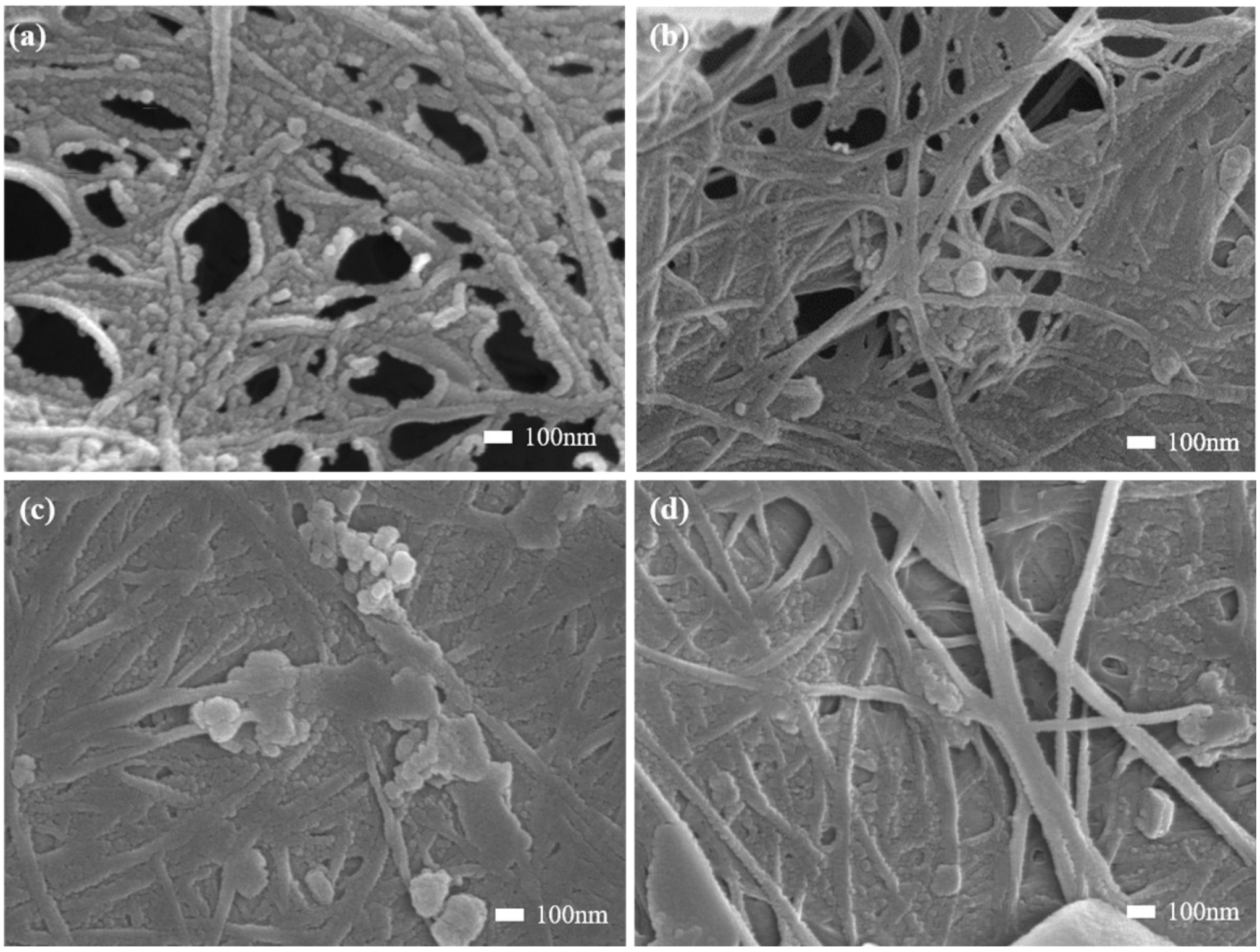

Figure S34. SEM images of (a, b) as-prepared HOF-21m@BC and (c, d) recovered HOF-21m@BC.

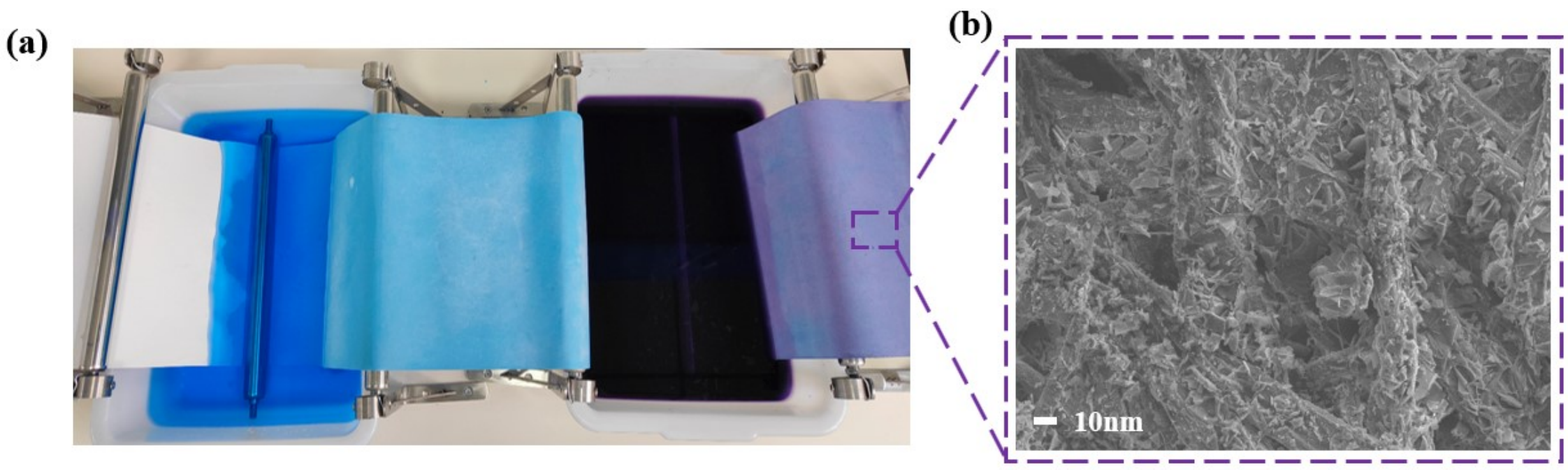

Figure S35. (a) Photographs of the continuous roll-to-roll fabrication of HOF-21m@cellulose membranes. (b) SEM image showing the uniform surface morphology of the HOF-21m@cellulose membrane.

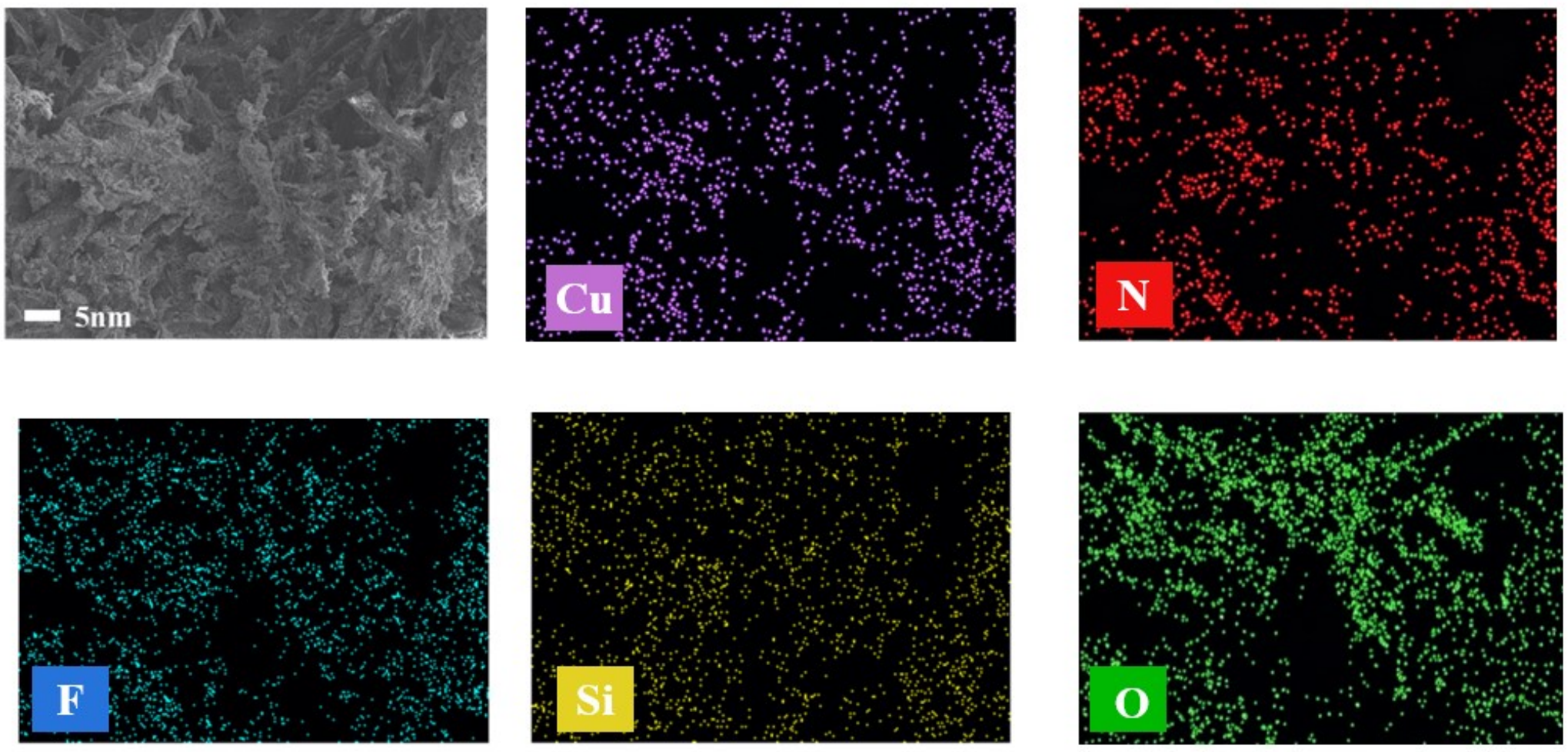

Figure S36. EDX elemental mapping of the HOF-21m@cellulose composite membrane, confirming the uniform distribution of characteristic elements and successful incorporation of HOF-21m into the cellulose framework.

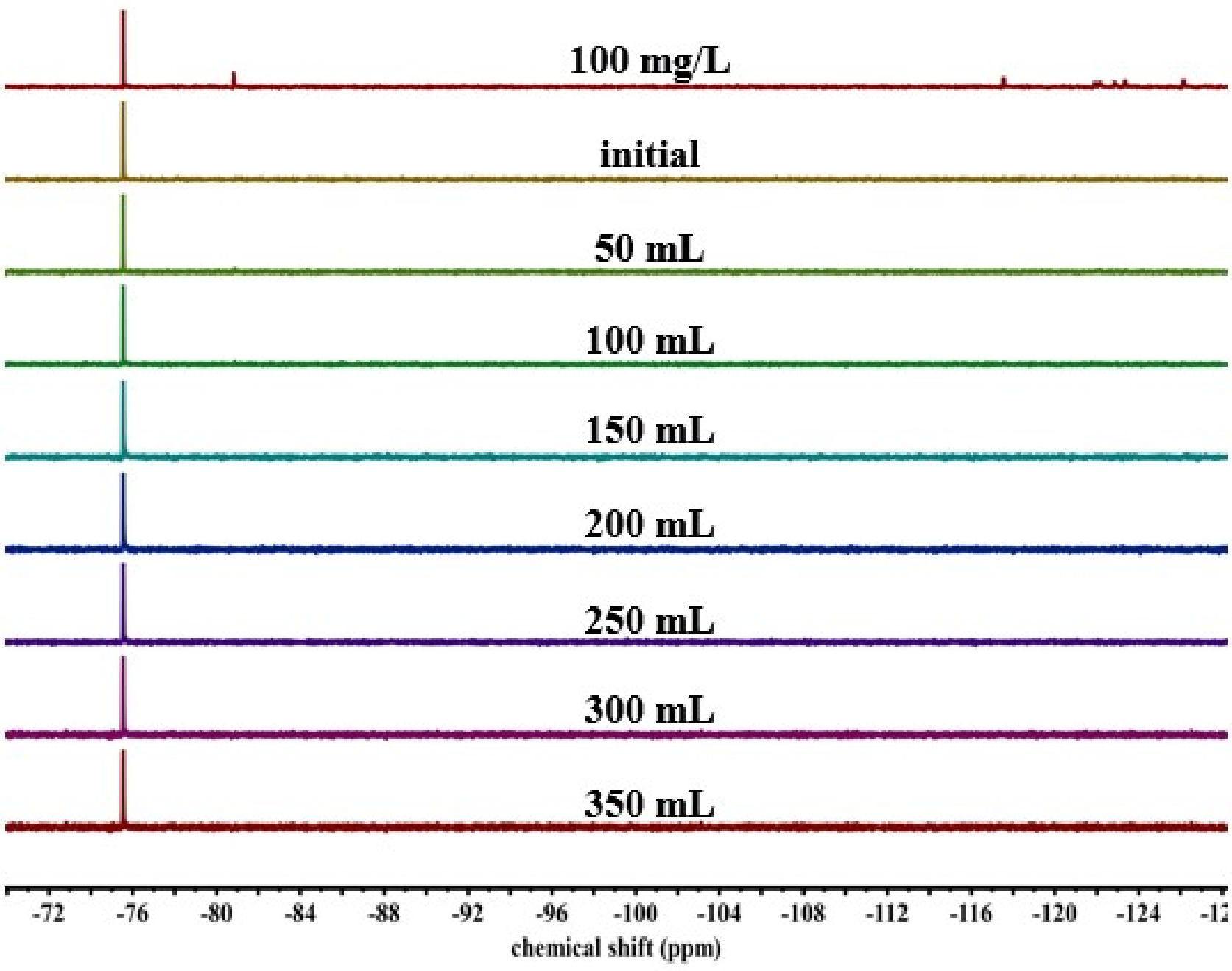

Figure S37. ^{19}F NMR spectra (396 MHz, 298K, H_2O:D_2O=9:1) of PFOA solutions filtered through the HOF-21m@cellulose membrane. Trifluoroacetic acid was used as an internal standard. Membrane diameter = 5.4 cm.

Table S1. Langmuir and Freundlich parameters derived from plots of the PFOA binding isotherm.

Langmuir Fit		Freundlich Fit		
Q_m (mg/g)	R^2	K_F(mg/g)(L/mg)$^{1/n}$	n	R^2
(6.57±0.38) x 10^3	0.993	72.51±18.56	1.70	0.975

Table S2. Concentration of Cu^{2+} before and after adsorption of PFOA.

Concentration of Cu^{2+} before adsorption	Concentration of Cu^{2+} before adsorption
8 ug/L	8.6±0.3 ug/L

Table S3. Comparison of the adsorption performance of HOF-21m with other representative adsorbent materials reported for PFOA removal, including MOFs, COFs, POPs, molecular cages, and activated carbon.

Materials	type	Q_m (mg g^{-1})
Fe-BTC[5]	MOFs	418
ZIF-L[6]	MOFs	496
MIL-53-Al-BPDC[7]	MOFs	220
MIL-101-Cr-QDMEN[8]	MOFs	768
Uio-67[9]	MOFs	743
Uio-66-(F4)[10]	MOFs	467
PCN-1002[11]	MOFs	632
PCN-999[12]	MOFs	1089
PAF-1-NDMB[13]	POPs	2000
β-CD-1[14]	POPs	457
F-Cage 2[15]	Cages	45
β-CD-COFs[16]	COFs	755
COF-300-dimethyl[17]	COFs	250
Cys-COF[18]	COFs	580
chitosan/F-COF[19]	COFs	6177
HOF-21m (this work)	HOFs	$(6.57\pm0.38) \times 10^3$

Table S4 Comparison of PFOA rejection and water permeance for HOF-21m@BC and representative membranes reported for PFAS removal, including RO, NF, and modified composite membranes.

Materials	concentration	Water permeance ($L\ m^{-2}\ h^{-1}\ bar^{-1}$)	Rejection (%)	Reference
MOF-PAm	50 μg/L	21.4	92.5	Ref[20]
MOFm	50 μg/L	60.6	51.3	
PAm	50 μg/L	28.8	64.5	
NF270	50 μg/L	17.2	93.7	
NF90	100 μg/L	10.5	65.1	Ref[21]
NFG	100 μg/L	12.3	60.2	
PIP	1000 μg/L	8.0	92.2	Ref[22]
PIP/BP	1000 μg/L	11.1	90.2	
SC0.2	50 μg/L	11.5	89.0	Ref[23]
NE70	1 μg/L	4.9	71.0	Ref[24]
ESPA-2540	60 μg/L	5.9	99.0	Ref[24]
ILIP-10	20 μg/L	11.0	80.6	Ref [25]
ZIF-L/PEI	200 μg/L	47.56	95.85	Ref[26]
TpPa-SO_3H	1000 μg/L	37.5	95.0	Ref[27]
HOF-21m@BC	100000 μg/L	86.3	99.9	This work

Table S5. Cost comparison of HOF-21m@cellulose membranes with NF, RO, and MOF/COF-based membranes.

Material	Type	Cost (dollar/m^2)	1/cost	Manufacturer/Ref
PSF	NF	248.9	0.00402	Safelab
Pam	NF	205.0	0.00488	Dow FilmTec
DK8040F-30D	NF	39.1	0.02551	GE Osmonics
NF270	NF	25.4	0.03936	Dow FilmTec
NF90	NF	20.9	0.04763	Hydranautics
ESPA2-8040	RO	96.2	0.01038	Hydranautics[22]
BW30-365	RO	13.6	0.0734	Dow FilmTec[28]
LFC1-365	RO	17.1	0.0585	Hydranautics[28]
LFC3-LD-4040	RO	8.9	0.11209	Hydranautics[28]
ILIP-10	MOF modified membrane	207.9	0.00481	Ref[25]
MOF-PAm	MOF modified membrane	206.8	0.00483	Ref[20]
PAS5	MOF modified membrane	146.4	0.00683	Ref[29]
ZIF-L/PEI	MOF modified membrane	250.7	0.00399	Ref[26]
TaPa-SO_3H	COF modified membrane	79.4	0.0126	Ref [27]
HOF-21m@cellulose	HOF modified membrane	3.5	0.28023	This work

References

1. Y. He, D. Luo, V. M. Lynch, M. Ahmed, J. L. Sessler and X. Chi, *Chem*, 2023, **9**, 93–101.
2. Z. Bao, D. Xie, G. Chang, H. Wu, L. Li, W. Zhou, H. Wang, Z. Zhang, H. Xing, Q. Yang, M. J. Zaworotko, Q. Ren and B. Chen, *J. Am. Chem. Soc*, 2018, **140**, 4596–4603.
3. Y. S. Ho and G. McKay, *Process Biochem*, 1999, **34**, 451–465.
4. T. Lu and F. Chen, *Journal of Computational Chemistry*, 2012, **33**, 580–592.
5. Y. Yang, Z. Zheng, W. Ji, J. Xu and X. Zhang, *J. Hazard. Mater*, 2020, **395**, 122686.
6. M.-J. Chen, A.-C. Yang, N.-H. Wang, H.-C. Chiu, Y.-L. Li, D.-Y. Kang and S.-L. Lo, *Microporous and Mesoporous Materials*, 2016, **236**, 202–210.
7. C. Zhao, Y. Xu, F. Xiao, J. Ma, Y. Zou and W. Tang, *Chem. Eng. J*, 2021, **406**, 126852.
8. K. Liu, S. Zhang, X. Hu, K. Zhang, A. Roy and G. Yu, *Environ. Sci. Technol*, 2015, **49**, 8657–8665.
9. K. Sini, D. Bourgeois, M. Idouhar, M. Carboni and D. Meyer, *Materials Letters*, 2019, **250**, 92–95.
10. K. Sini, D. Bourgeois, M. Idouhar, M. Carboni and D. Meyer, *New J. Chem*, 2018, **42**, 17889–17894.
11. R.-R. Liang, Y. Yang, Z. Han, V. I. Bakhmutov, J. Rushlow, Y. Fu, K.-Y. Wang and H.-C. Zhou, *Adv. Mater*, 2024, **36**, 2407194.
12. R.-R. Liang, S. Xu, Z. Han, Y. Yang, K.-Y. Wang, Z. Huang, J. Rushlow, P. Cai, P. Samorì and H.-C. Zhou, *J. Am. Chem. Soc*, 2024, **146**, 9811–9818.
13. X. Liu, C. Zhu, J. Yin, J. Li, Z. Zhang, J. Li, F. Shui, Z. You, Z. Shi, B. Li, X.-H. Bu, A. Nafady and S. Ma, *Nat. Commun*, 2022, **13**, 2132.
14. A. Yang, C. Ching, M. Easler, D. E. Helbling and W. R. Dichtel, *ACS Materials Lett*, 2020, **2**, 1240–1245.
15. Y. He, J. Zhou, Y. Li, Y.-D. Yang, J. L. Sessler and X. Chi, *J. Am. Chem. Soc*, 2024, **146**, 6225–6230.
16. W. Wang, H. Shao, S. Zhou, D. Zhu, X. Jiang, G. Yu and S. Deng, *ACS Appl. Mater. Interf*, 2021, **13**, 48700–48708.
17. A. N. Zeppuhar, D. S. Rollins, D. L. Huber, E. A. Bazan-Bergamino, F. Chen, H. A. Evans and M. K. Taylor, *ACS Appl. Mater. Interf*, 2023, **15**, 52622–52630.
18. J. Huang, Y. Shi, J. Xu, J. Zheng, F. Zhu, X. Liu and G. Ouyang, *Adv. Funct. Mater*, 2022, **32**, 2203171.
19. C. He, Y. Yang, Y.-J. Hou, T. Luan and J. Deng, *Sep. Purif. Technol*, 2022, **294**, 121195.
20. K. Zhang, P. Cheng, Y. Liu and S. Xia, *Water Res*, 2024, **265**, 122276.
21. M. Li, F. Sun, W. Shang, X. Zhang, W. Dong, Z. Dong and S. Zhao, *Chem. Eng. J*, 2021, **406**, 126814.
22. C. Boo, Y. Wang, I. Zucker, Y. Choo, C. O. Osuji and M. Elimelech, *Environ. Sci. Technol*, 2018, **52**, 7279–7288.
23. Z. Wang, Y. Gao, J. Zhang, Z. Peng, X.-m. Wang, S. Liu and X. Huang, *Sep. Purif. Technol*, 2023, **322**, 124329.
24. C. J. Liu, T. J. Strathmann and C. Bellona, *Water Res*, 2021, **188**, 116546.
25. Y. Zhao, X. Tong, J. Kim, T. Tong, C.-H. Huang and Y. Chen, *Environ. Sci. Technol*, 2022, **56**, 5849–5859.
26. Y. Bi, X. Meng, Z. Tan, Q. Geng, J. Peng, Q. Yong, X. Sun, M. Guo and X. Wang, *Sci. Total.*

Environ, 2024, **925**, 171727.
27. T.-T. Nguyen, X. Min, W. Xia, Z. Qiang, R. S. Khandge, H.-K. Yu, J.-W. Wang, Y. Wang and X. Ma, *J. Memb. Sci*, 2024, **705**, 122925.
28. C. Y. Tang, Q. S. Fu, C. S. Criddle and J. O. Leckie, *Environ. Sci. Technol*, 2007, **41**, 2008–2014.
29. J. Zhang, Z. Huang, L. Gao, S. Gray and Z. Xie, *Sci. Total. Environ*, 2022, **806**, 151207.
